# Recent advances in 2D van der Waals magnets: Detection, modulation, and applications

**DOI:** 10.1016/j.isci.2023.107584

**Published:** 2023-08-11

**Authors:** Ping Liu, Ying Zhang, Kehan Li, Yongde Li, Yong Pu

**Affiliations:** 1 School of Science & New Energy Technology Engineering Laboratory of Jiangsu Province, Nanjing University of Posts and Telecommunications, Nanjing 210023, China; 2 Department of Materials Science & Engineering, CAS Key Lab of Materials for Energy Conversion, University of Science and Technology of China, Hefei 230026, China

**Keywords:** Nanomaterials, magnetism, condensed matter physics

## Abstract

The emergence of two-dimensional (2D) van der Waals magnets provides an exciting platform for exploring magnetism in the monolayer limit. Exotic quantum phenomena and significant potential for spintronic applications are demonstrated in 2D magnetic crystals and heterostructures, which offer unprecedented possibilities in advanced formation technology with low power and high efficiency. In this review, we summarize recent advances in 2D van der Waals magnetic crystals. We focus mainly on van der Waals materials of truly 2D nature with intrinsic magnetism. The detection methods of 2D magnetic materials are first introduced in detail. Subsequently, the effective strategies to modulate the magnetic behavior of 2D magnets (e.g., Curie temperature, magnetic anisotropy, magnetic exchange interaction) are presented. Then, we list the applications of 2D magnets in the spintronic devices. We also highlight current challenges and broad space for the development of 2D magnets in further studies.

## Introduction

Electrons have two intrinsic properties: spin and charge. The development of semiconductor technology based on the charge of electrons triggered the third generation of the industrial revolution, which greatly changed the way of human life. Spintronics devices use electron spin as an information carrier, which is considered as one of the core technologies of the next information industry revolution.^1^^,^^2^ The macroscopic manifestation of electron spin is magnetism, such as ferromagnetism (FM), ferrimagnetism (FIM), and antiferromagnetism (AFM).^3^^,^^4^ The research of magnetism is the elementary building block in the spintronic application. With the development of the miniaturization of electronic devices, low-dimensional magnetism becomes a research hotspot.^5^ In the early stages, nano-scaled magnetic materials with atomic thickness are usually prepared in traditional thin film technology, such as electron beam evaporation, magnetron sputtering, molecular beam epitaxy, pulsed laser deposition, thermal evaporation,^6^^,^^7^^,^^8^^,^^9^ etc. The essence of these techniques is to deposit three-dimensional (3D) magnetic block material on the substrate to obtain magnetic thin films with two-dimensional (2D) geometric morphology. The magnetism of films strongly depends on the substrate and inevitably has interface defects due to the stress, which is hard to realize atomic magnetic devices.

The successful exfoliation of graphene in 2004 has led to a great impact on material science and low-dimensional physics.^10^ Due to the absence of chemical hanging bonds between the surface and the intralayer, the influence of interface defects can be effectively eliminated.^11^ Thus, 2D layered materials exhibit excellent physicochemical properties and electronic performance.^12^ Therefore, intensive efforts have been made to induce magnetism in 2D van der Waals layered materials.^13^ Various strategies such as doping or adsorption,^14^^,^^15^ defect engineering,^16^^,^^17^ and proximity effect^18^ at the interface have introduced effective macroscopic magnetic moments in 2D materials. However, such induced magnetism is weak, localized, and unable to modulate. Moreover, the scattering effect from the magnetic impurities or defects hinders the development of practical device applications. Realizing long-range magnetic order in 2D limit still faces great challenges.

**Figure 1:**
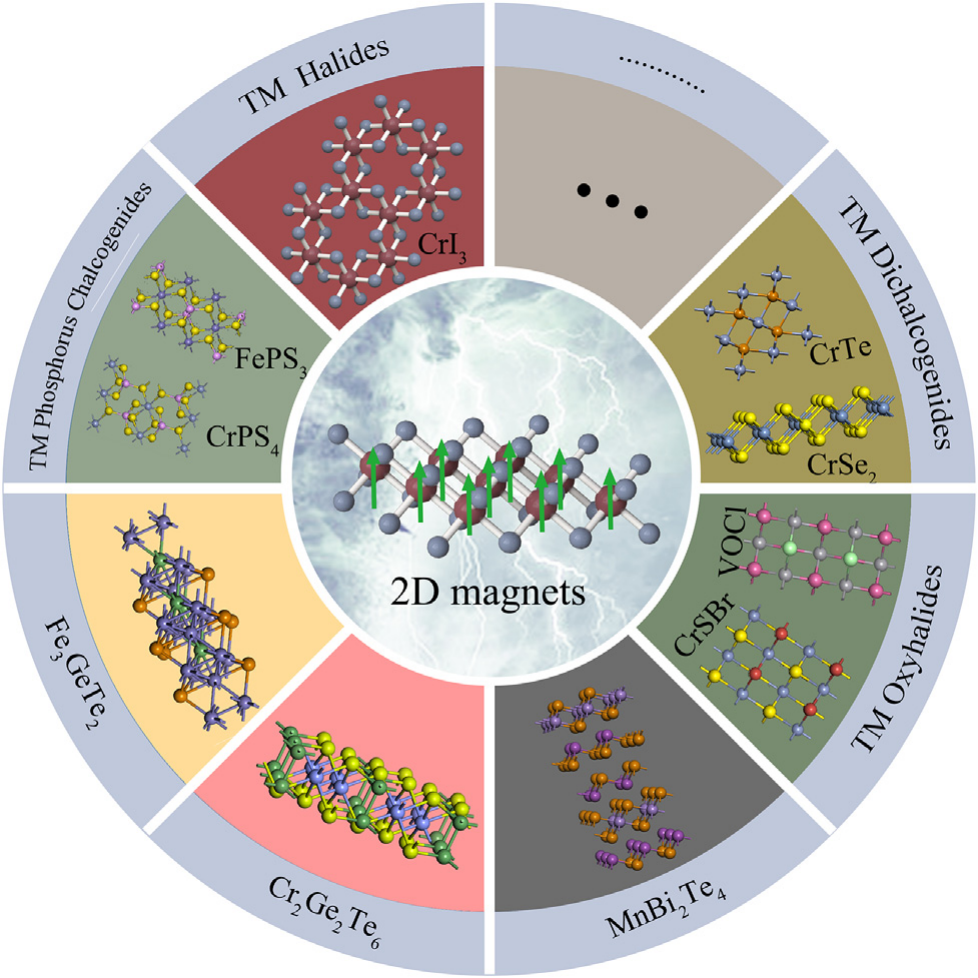
A typical family of 2D magnetic crystals The synthesis methods of 2D magnetic materials mainly include chemical vapor transport (CVT), flux method, mechanical exfoliation, molecular beam epitaxy (MBE), and chemical vapor deposition (CVD).^41^ To date, the reported high-quality vdWs magnetic crystals are mostly obtained by the CVT and flux method. Then, thin flakes of 2D magnets such as transition metal halides Cr×_3_, CrSi(Ge)Te_3_, Fe_3_Ge(Ga)Te_2_, MPX_3_, CrPS_4_, and CrOCl, are fabricated through mechanical exfoliation from their bulk crystals. In order to obtain monolayer and large-scale magnetic samples, researchers have improved mechanical exfoliation by using Au or Al_2_O_3_ as adhesive layers.^42^^,^^43^ Mechanical exfoliation can obtain high-quality samples and enable the direct exploration of the intrinsic magnetic properties of 2D magnets. However, it is extremely time-consuming and laborious, which is unable to meet practical industrial production. MBE is an effective method for growing 2D magnetic films with a controllable thickness. For example, 2D ferromagnetic Fe_*x*_GeTe_2_, CrTe_2_, MnSe_2_, and CrBr_3_ are successfully synthesized by MBE.^40^^,^^44^^,^^45^ However, it also faces challenges in high cost, low efficiency, and complicated process. CVD is considered as a suitable method to produce large-area 2D magnet materials, which is successfully verified in a series of binary transition metal chalcogenide magnets such as Cr_3_Te_4_,^46^ CrSe_2_,^28^ Cr_5_Te_8_.^47^ At present, the kinds of 2D magnetic nanosheet growth using CVD are limited and need to be developed.

According to Mermin–Wagner theory, spontaneous polarization does not exist in the isotropic 2D Heisenberg model at a finite temperature.^19^ Therefore, a prerequisite for the establishment of magnetic order in the 2D limit is that the strong magnetic anisotropy can open an energy gap in the spin-wave spectrum and suppresses thermal fluctuations. In 2016, the first stable antiferromagnetic ordering was reported in atomically thin magnetic vdWs FePS_3_ crystals through the Raman spectrum.^20^ Soon in 2017, the direct evidence of spontaneous magnetization in Cr_2_Ge_2_Te_6_ and CrI_3_ with the thickness down to the monolayer limit was revealed by the magneto-optic Kerr effect (MOKE),^21^^,^^22^ respectively. Encouraged by the demonstration of 2D magnetism in CrI_3_ and Cr_2_Ge_2_Te_6_, more and more vdWs magnets have been discovered, which provide an exciting research platform for understanding and regulating low-dimensional magnetism. At present, the reported 2D vdWs magnetic materials mainly include transition metal halides M×_3_ or MX_2_ (M = V, Cr; X = Cl, Br, I),^23^^,^^24^^,^^25^ binary transition metal chalcogenides such as MX, M_2_X_3_, M_5_X_8_, MX_2_ (M = Cr, V; X = S, Se, Te),^26^^,^^27^^,^^28^ transition metal phosphorus chalcogenides MPX_3_ (M = Fe, Co, Ni, Mn; X = S, Se)^29^ and CrPS_4_,^30^ Mn (Sb)-Bi-Te family,^31^^,^^32^^,^^33^ Fe_3_Ge(Ga)Te_2_ family,^34^^,^^35^^,^^36^ MXY (M = Cr, V; X = O, S, Se, Te; Y= Cl, Br, I),^37^^,^^38^^,^^39^ etc, as shown in Figure 1. More exciting, room temperature ferromagnets like CrTe_2_ and Fe_3_GaTe_2_ have been reported very recently.^36^^,^^40^ And we expect more and more vdWs magnets to be found with striking magnetic properties.

The 2D van der Waals magnets exhibit intrinsic and thickness-dependent magnetic properties without the substrate effect, which is distinct from traditional 3D magnetic materials. Benefiting from the 2D layered structure, 2D magnetism is more sensitive to external stimuli such as mechanical stress, pressure, electrical field, light, and interface coupling, which can be effectively modulated.^48^ More interestingly, unprecedented and exotic physical phenomena have emerged in 2D magnetic heterostructures by stacking 2D magnets with dissimilar materials, offering new opportunities for designing advanced spintronic devices.^49^ In this review, we give rise to an overview of recent advances in 2D van der Waals magnets. Firstly, we summarized the various techniques for detecting magnetic ground states in 2D systems. Secondly, we introduce efficient strategies for modulating the magnetic properties of vdWs magnets. Thirdly, we present the recent progress of 2D magnets in spintronics devices and magnetic storage. Finally, we discuss the challenges and perspectives of future development of 2D magnets. We also highlight the significant prospect in the research of information devices and low-dimensional physics.

### Detection of 2D magnetism

The 2D vdWs magnetic materials are mostly prepared by mechanical exfoliation, which are relatively small nanoflakes. Thus, traditional magnetic characterization technologies such as superconducting quantum interference device (SQUID) magnetometry, vibrating sample magnetometer (VSM), and neutron diffraction experiments, failed to detect the magnetism of 2D materials. These techniques are restricted to the weak signal and small size of the micrometer-scale 2D samples. Therefore, several special techniques have been employed to explore the magnetism of 2D materials. Herein, we will give a comprehensive introduction to the current techniques used to detect the magnetic ground state of 2D magnets.

### Optical techniques

#### Magneto-optical Kerr effect (MOKE)

The experimental observation of intrinsic ferromagnetism in the first two van der Waal materials down to the 2D limit was demonstrated by MOKE measurements. The MOKE refers to changes in the polarization of reflected light after the interaction between linearly polarized light (consists of left- and right-handed circularly polarized light) and magnetic materials.^50^ By measuring the polarizability or signal strength of the reflected light, magnetic information can be obtained such as the magnetic domain, magnetization process, and magnetization curves. Because the MOKE signal strength is proportional to the magnetization, hysteresis loops can be obtained by measuring the MOKE signal varying with the magnetic field. Compared with traditional magnetic measurement methods, MOKE takes the advantages of non-destructive, high spatial resolution, and sensitivity to the surface and interface, which is beneficial to magnetic materials at the nanoscale.

**Figure 2:**
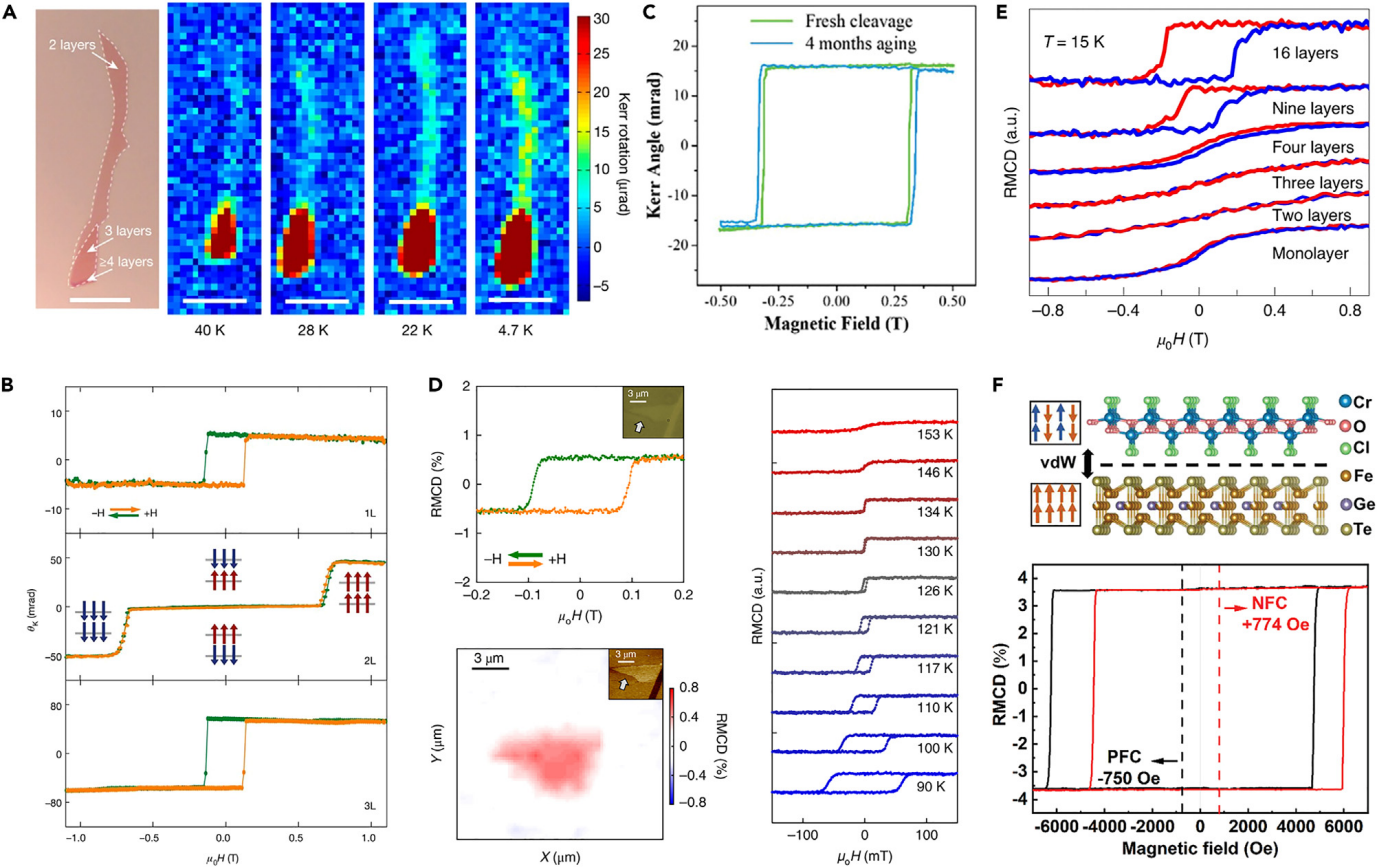
Detection of 2D magnetism using MOKE and RMCD (A) Direct observation of ferromagnetism in bilayer Cr_2_Ge_2_Te_6_ by mapping the Kerr rotation signal. Scale bars: 10 μm. Reproduced with permission from Gong et al.^22^ Copyright © 2017, Springer Nature. (B) Layer-dependent magnetic ordering in atomically thin CrI_3_. Reproduced with permission from Huang et al.^21^ Copyright © 2017, Springer Nature. (C) MOKE signal of air-stable Ta_3_FeS_6_ nanosheet acquired before and after 4 months. Reproduced with permission from Su et al.^51^ Copyright © 2020, Wiley-VCH. (D) RMCD measurements of monolayer Fe_3_GeTe_2_. Reproduced with permission from Fei et al.^54^ Copyright © 2018, Springer Nature. (E) Thickness-dependent magnetic properties of CrSe_2_ nanosheets at 15 K. Reproduced with permission from Li et al.^28^ Copyright © 2021, Springer Nature. (F) Schematic diagram of the device structure of the FGT/CrOCl heterostructure and the exchange bias effect revealed by field-dependent RMCD signals. Reproduced with permission from Zhang et al.^56^ Copyright © 2022, Wiley-VCH.

In 2017, Zhang’s group investigated the ferromagnetic properties of thin layer Cr_2_Ge_2_Te_6_ by magneto-optical Kerr technology. Figure 2A shows the magneto-optical Kerr signal distribution of double-layer Cr_2_Ge_2_Te_6_ at different temperatures. The experimental results show that the magnetic signal can only be detected if Cr_2_Ge_2_Te_6_ sample is more than two layers thick at the temperature of 40 K.^22^ When the temperature drops to 4.7 K, the bilayer samples show an obvious ferromagnetic signal. The Curie temperature of Cr_2_Ge_2_Te_6_ exhibits a monotonic increase with increasing thickness, from 30 K of the bilayer to 68 K of a bulk sample. At the same time, similar investigations on intrinsic ferromagnetism of monolayer CrI_3_ have also been demonstrated.^21^ The ferromagnetism of CrI_3_ is strongly dependent on the layers (Figure 2B). Monolayer, trilayer, and bulk CrI_3_ exhibit ferromagnetism, while the bilayer CrI_3_ shows antiferromagnetism. When the external magnetic field increases to 0.65 T, the anti-ferromagnetic to ferromagnetic transition occurs in the bilayer CrI_3_. Su et al. revealed the thickness-tunable ferromagnetism in 2D Ta_3_FeS_6_ nanosheets by MOKE technique.^51^ It is found that the magneto-optical Kerr signal of the Ta_3_FeS_6_ nanosheet shows negligible degradation after four months (Figure 2C), indicating high stability under an atmospheric environment. Similar investigations were performed on the 2D Cr_5_Te_8_.^52^ In addition to detecting the ferromagnetic signals, MOKE is an effective tool to obtain the magnetization reversal information of 2D magnets. Liu et al. observed an antisymmetric magnetoresistance (MR) in a vdWs Fe_3_GeTe_2_ (FGT) flake with a step terrace, which is due to the unsynchronized magnetization switching between the step structure.^53^ In order to confirm that, MOKE is employed to image the domain structure in the stepped FGT flake by scanning an external magnetic field. Through the micro-MOKE images of FGT flake, the opposite magnetization direction can be clearly found on both sides of the step. With the help of MOKE, we can obtain the magnetic properties and magnetic dynamics characteristics of 2D magnetic materials at the nanoscales.

#### Magnetic circular dichroisms (MCD)

MCD describes the difference of absorption toward left-handed and right-handed light when incident polarized light passes through the sample under an external magnetic field. If the materials have a long-range magnetic order, it will lead to differences in the absorption of left-handed and right-handed light. The magnetic state of the material can be inferred from the amplitude and phase of the outgoing elliptically polarized light.

Fei et al.^54^ revealed a crossover from 3D to 2D Ising ferromagnetism of Fe_3_GeTe_2_ for thicknesses less than 4 nm (five layers) based on the remnant reflective magnetic circular dichroism (RMCD) signals as a function of temperature. It is found that the Curie exhibits a fast drop from 207 K (5 layers) to 130 K in the monolayer (Figure 2D). Moreover, the hysteresis loops are different for thinner and thicker Fe_3_GeTe_2_ samples. When the temperature is between 185 K and 210 K, the hysteresis loop of thicker Fe_3_GeTe_2_ exhibits complex behavior, which is related to the labyrinthine domains. For the thinner Fe_3_GeTe_2_ (3.2 nm), the hysteresis loop maintains a rectangular shape below *T*_C_, indicating a single ferromagnetic phase. Song et al. explored the magnetic textures in the twisted bilayer CrI_3_ with the help of RMCD.^55^ The coexistence of antiferromagnetic (AFM) and ferromagnetic (FM) domains with disorder-like spatial patterns were observed through the RMCD mapping.

Li et al. used polar RMCD to reveal layer-dependent magnetic properties of CrSe_2_ nanosheets.^28^ In contrast to FGT, CrSe_2_ nanosheets are weak ferromagnets with nearly zero coercive field from monolayer to tri-layers and become stronger ferromagnets with increasing thicknesses (Figure 2E). In addition to the detection of ferromagnetism, the RMCD are widely used to explore various magnetic effect, such as the exchange bias effect. As shown in Figure 2F, Zhang et al. established FGT/CrOCl heterostructure and used the RMCD hysteresis loops to determine the coercive bias field as a function of layers of ferromagnets or antiferromagnets.^56^ In addition to studying the magnetic properties of micro-samples, other magnetic information such as atomic spin, orbital magnetic moments, and electronic transition can also be detected through the MCD technique.

#### Raman spectroscopy

Raman spectroscopy is an effective and non-destructive way to detect the magnetism of 2D materials, especially for anti-ferromagnetism. The antiferromagnetic materials exhibit zero net magnetic moment due to the antiparallel arrangement of spins, which is more difficult to detect the antiferromagnetic ordering. The long-range magnetic ordering of materials can generate magnetic excitations or modulate the phonon vibration modes. Thus, the magnetic ordering in 2D materials can be deduced by analyzing the Raman spectrum related to magnon scattering and spin-phonon coupling, such as the intensity and location of the Raman peaks.^57^

**Figure 3:**
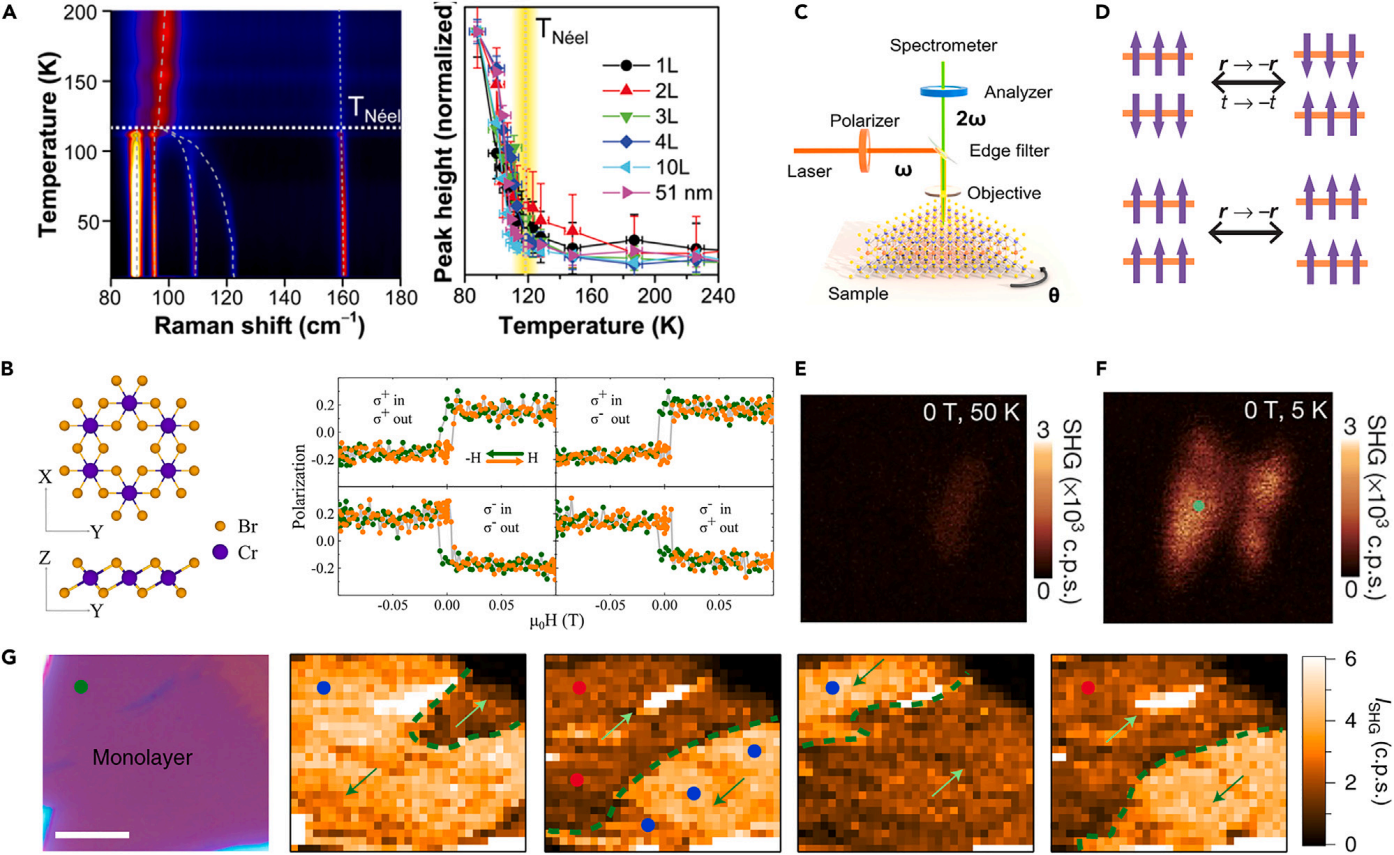
Detection of 2D magnetism using Raman spectra, photoluminescence, and SHG measurements (A) Temperature dependence of Raman peaks for different thicknesses in atomically thin FePS_3_. Reproduced with permission from Lee et al.^20^ Copyright © 2016, American Chemical Society. (B) The crystal structure of CrBr_3_ and its polarization as a function of the magnetic field. Reproduced with permission from Zhang et al.^62^ Copyright © 2019, American Chemical Society. (C) Schematic image of SHG testing. Reproduced with permission from Xu et al.^65^ Copyright © 2022, American Chemical Society. (D) Schematics of two-layered antiferromagnetic and ferromagnetic states of a CrI_3_ bilayer. (E and F) The SHG intensity images when the bilayer CrI_3_ is nonmagnetic (0 T, 50 K), and (F) antiferromagnetic (0 T, 5 K). (D, E, and F) are reproduced with permission from Sun et al.^66^ Copyright © 2019, Spring Nature. (G) SHG intensity mapping of the monolayer MnPSe_3_ sample at 5 K after different thermal cycles. Scale bar: 10 μm. Reproduced with permission from Ni et al.^67^ Copyright © 2021, Spring Nature.

Recently, Raman spectroscopy has revealed the magnetic ordering of several 2D antiferromagnets with Ising,^20^ XY,^58^ and Heisenberg-type models.^59^ Lee et al. confirmed the Néel temperature of Ising-type FePS_3_ in a few layers was 118 K by Raman spectroscopy. There is a Raman vibration mode in FePS_3_, which arises from zone-folding effect due to the antiferromagnetic ordering. Therefore, this peak is regarded as a Raman signature of antiferromagnetic ordering. As shown in Figure 3A, the variation of Raman peak intensity as a function of temperature is consistent with the magnetic transition of FePS_3_. The Néel temperature remains almost unchanged even though the thickness is down to monolayer. Jin et al. explored the thickness-dependent 2D ferromagnetism in CrI_3_ by polarized micro-Raman spectroscopy.^60^ Two typical Raman modes at 2.28 THz and 3.75 THz were identified as single-magnon excitation of CrI_3_. The integrated intensity of magnons as a function of temperature was fitted by I.I. ∝ $\sqrt{T_{C}-T}$. By plotting the thickness dependence of both spin wave intensities, the phase diagram of 2D CrI_3_ was obtained and revealed the transition temperature of ∼45 K. In addition to the identification of phonon or magnon excitation, the Raman signals related to the magnon-phonon hybridization can also be marked as a fingerprint to confirm the magnetic state.^61^ Mai et al. observed the signals of hybridization of two-magnon excitations with two phonons in the Raman spectrum. By measuring the phonons’ lifetime, the magnon lifetime reduction was identified in 2D MnPSe_3_. Raman spectroscopy has been established as an important tool to investigate the magnetic ordering and magnetic excitations in 2D magnets.

#### Photoluminescence (PL) measurement

PL measurement has been used to reveal the magnetic order state in some 2D semiconductor van der Waals materials. The helicity of photoluminescence in 2D materials is strongly related to its intrinsic magnetic ordering. To reveal the ferromagnetism of atomically thin CrBr_3_, Zhang et al. analyzed the PL through the excitation of the circularly polarized light and detection of the left (or right) circularly polarized light.^62^ The zero polarization is identified as the average PL intensity of fully spin up and down states of CrBr_3_. Then, polarization as a function of the magnetic field was obtained as shown in Figure 3B, exhibiting a characteristic hysteresis loop. Seyler et al. observed spontaneous left and right circularly polarized luminescence at zero fields in monolayer CrI_3_ by incident linearly polarized light, demonstrating the out-of-plane magnetic ordering.^63^ By plotting the PL circular polarization as a function of temperature, the transition temperature of CrI_3_ is determined to be 45 K, which is consistent with the previous MOKE results. While for bilayer CrI_3_, the circular polarization as a function of the magnetic field shows the vanishing out-of-plane magnetization at a magnetic field range of +0.65 T and −0.65 T, suggesting the antiferromagnetic coupling. More than CrX_3_, other 2D antiferromagnetic materials such as NiPS_3_ exhibit a spin-induced linear polarization of photoluminescence.^64^ Compared to the traditional magnetic detection technologies, the PL measurement offers an easy, fast, nondestructive strategy to determine the Néel vector orientation in NiPS_3_.

#### Second harmonic generation (SHG) measurement

Second harmonic generation is another powerful technique to detect magnetism. SHG is the simplest second-order nonlinear process, in which two photons of the same frequency (ω) interact with the nonlinear optical material and radiate a photon of double frequency (2*ω*) (Figure 3C).^65^ SHG has a high sensitivity to symmetry breaking of materials, which allows the detection of symmetry-related physical phenomena, such as phase transition, ferroelectric, and ferromagnetic order. This enables SHG measurements feasible to detect the 2D magnetism. The MOKE and RMCD measurements have demonstrated the bilayer CrI_3_ is interlayer AFM coupling and remains a centrosymmetric lattice structure. Thus, the *i*-type SHG signal should be forbidden in bilayer CrI_3_ (Figure 3D). However, a strong SHG signal is detected in bilayer CrI_3_ at the antiferromagnetic state, which originated from the magnetic structure that can break both time-reversal and spatial inversion symmetries and induce the *c*-type SHG signal.^66^ The SHG intensity mapping of bilayer CrI_3_ in the nonmagnetic and antiferromagnetic states (Figures 3E and 3F) demonstrated the correlation between the interlayer antiferromagnetic coupling and structural information. The polarization-dependent SHG measurements reveal the monoclinic stacking structure of bilayer CrI_3_, which provide direct experimental evidence of structural information related to the antiferromagnetic coupling. In addition to the detection of AFM order and its transition temperature, more magnetic information such as types of the magnetic domain can also be obtained by SHG microscope. Ni et al. detect long-range Néel AFM order and Ising type Néel vector switching in the monolayer MnPSe_3_ by the spatially resolved SHG measurement.^67^ As shown in Figure 3G, SHG intensity mapping after different thermal cycles directly images the two antiferromagnetic domains with antiphase (180°). SHG also can be employed to characterize the emergence of ferromagnetic order in 2D magnets. CrSBr is an A-type antiferromagnet in bulk and transforms to a ferromagnet in monolayer limit. Lee et al.^68^ have successfully probed the presence of ferromagnetism in monolayer CrSBr by SHG. In addition to the above 2D materials, SHG is widely used in various 2D antiferromagnets including the MnBi_2_Te_4_,^69^ CrOCl,^37^ and MnPS_3_.^70^^,^^71^ All these experimental results suggest the SHG measurements are generically applicable to detecting 2D magnetism.

### Microscopy characterizations

Microscopy technology is a powerful tool to directly and dynamically record the magnetic domains, domain walls, and skyrmions from the microscopic level. Magnetic probing techniques based on electron microscopy can provide more accurate magnetic information at the nanoscale, which can better understand the magneto-transport behavior of spintronics devices and thus promote the development of magnetic information recording and reading technology.

#### Magnetic force microscopy (MFM)

**Figure 4:**
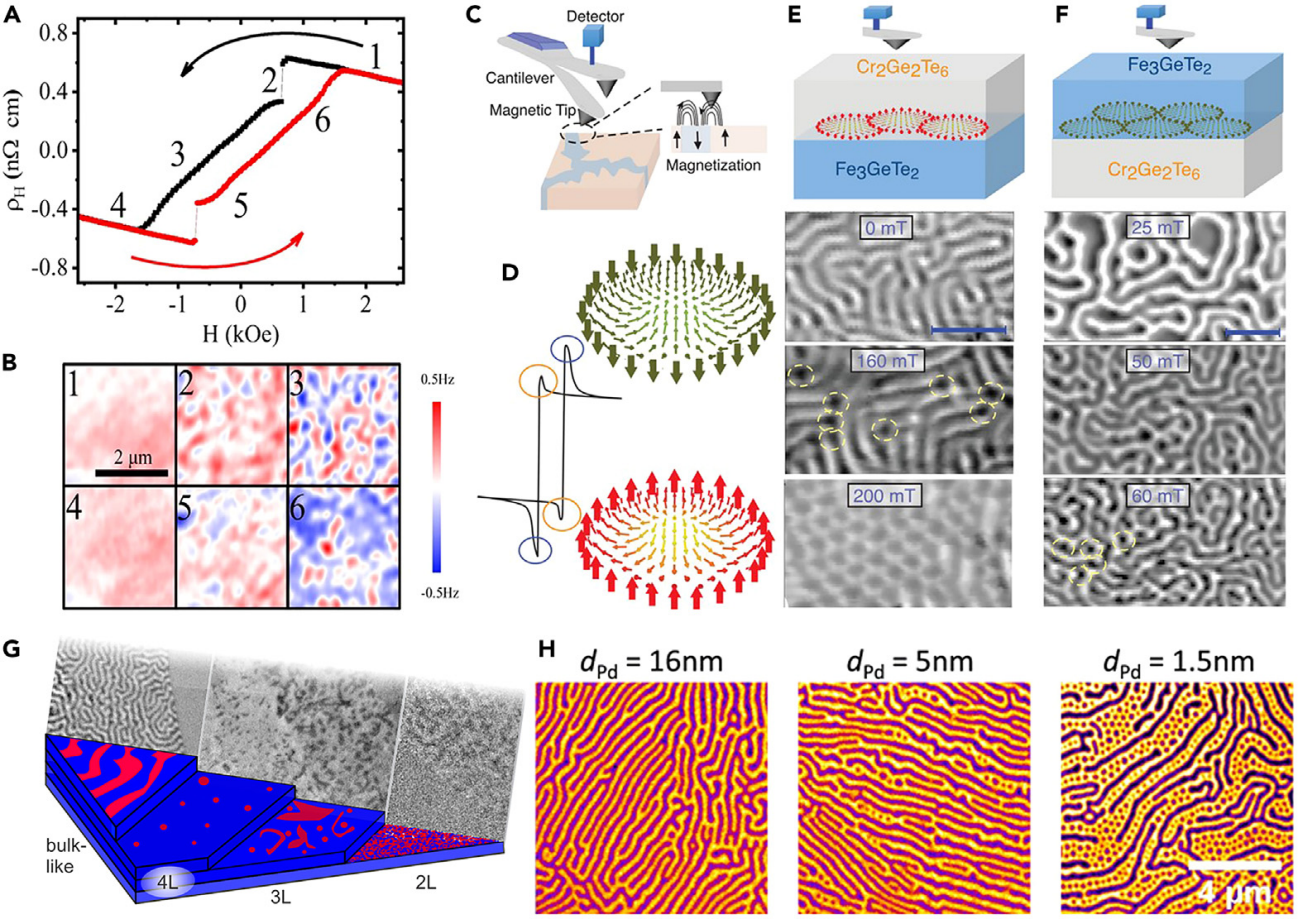
The magnetism detection through MFM and XPEEM measurements (A) Hall hysteresis loop of Cr_2_Ge_2_Te_6_/Pt heterostructure measured at 4 K. (B) MFM images at different magnetic fields marked in (A). Figures A and B are reproduced with permission from Lohmann et al.^72^ Copyright © 2019, American Chemical Society. (C) Schematic illustration of MFM measurements of Fe_3_GeTe_2_/Cr_2_Ge_2_Te_6_ heterostructure. (D) Two sets of THE signals observed for temperatures lower than 60 K in the Fe_3_GeTe_2_/Cr_2_Ge_2_Te_6_ heterostructure. The blue and orange circles represent THE on the Fe_3_GeTe_2_ and Cr_2_Ge_2_Te_6_, respectively. (E) Skyrmion lattice observed on the Cr_2_Ge_2_Te_6_ side when Cr_2_Ge_2_Te_6_ layer is on top of the Fe_3_GeTe_2_ layer. Scale bar: 1 μm. (F) Skyrmions on the Fe_3_GeTe_2_ side when Fe_3_GeTe_2_ layer is on top of the Cr_2_Ge_2_Te_6_ layer. Scale bar: 1 μm. (C–F) are reproduced with permission from Wu et al.^73^ Copyright © 2022, Wiley-VCH. (G) Layer-dependent magnetic domains in atomically thin Fe_5_GeTe_2_. Reproduced with permission from Fujita et al.^75^ Copyright © 2022, American Chemical Society. (H) PEEM images of the Fe_3_GeTe_2_ magnetic domains on (Co/Pd)_n_ superlattice with different thicknesses of the Pd spacer layer. Reproduced with permission from Yang et al.^76^ Copyright © 2020, American Association for the Advancement of Science.

Magnetic force microscopy can resolve the magnetic field distribution near the surface of magnetic films below tens of nanometers. It is a powerful tool to obtain the domain distribution information of microparticles at the submicron scale. A recent effort to probe magnetism in insulating Cr_2_Ge_2_Te_6_ (CGT) by the anomalous Hall effect of adjacent Pt has been made with the help of MFM.^72^ The induced anomalous hall effect exhibits distinct hysteresis loops (Figure 4A), which indicated the nonuniform magnetization of the underlying Cr_2_Ge_2_Te_6_. The spatial variation of MFM signals is indicative of the magnetic domains. By the MFM imaging as shown in Figure 4B, the transition from the single-domain state to multiple domains was observed, which corresponds to the characteristics of the hysteresis loop. The abrupt drop of the anomalous Hall effect signal at state 2 is caused by the domain nucleation. When the magnetic field is reversed at state 3, the MFM signals show opposite contrast, suggesting the formation of opposite domains. The magnetic domain information captured by MFM can provide a better understanding of the magnetic transport phenomenon.

To further explore the origin of the topological Hall effect (THE) of the Fe_3_GeTe_2_/Cr_2_Ge_2_Te_6_ interface, the MFM was used to image the microscopic magnetic domain structure (Figure 4C).^73^ As shown in Figure 4D, two sets of kinks of THE signal are observed at the heterostructure Fe_3_GeTe_2_/Cr_2_Ge_2_Te_6_ of two ferromagnets, which are explained by the formation of two groups of magnetic skyrmions. To claim the above hypothesis and distinguish the origin of the skyrmions, the top and bottom FM layer stacking are designed in a different order. Figure 4E shows a skyrmion lattice with a size of ≈130 nm on the Cr_2_Ge_2_Te_6_ side when the magnetic field exceeds 160 mT at 20 K. While in a reversed structure, the skyrmion lattice starts to emerge on the Fe_3_GeTe_2_ side with a magnetic field of 50 mT at 100 K (Figure 4F). In a word, MFM is very beneficial and sensitive to the study of the microscopic magnetic distribution of 2D magnetic materials.

#### X-ray photoemission electron microscopy (XPEEM)

XPEEM technique is based on imaging the photoemitted secondary electrons from a sample irradiated by X-rays, which combines the high resolution of electron microscopy with the magnetic and chemical sensitivity of soft X-ray absorption. It can provide surface and subsurface properties of materials by several contrast mechanisms such as topography, chemical, elemental, polarization, etc. Therefore, XPEEM is a powerful tool to characterize the element-selective magnetic domains in ultrathin vdWs magnets. In an early work, XPEEM is employed for real-space imaging of 2D itinerant ferromagnet Fe_3_GeTe_2_.^74^ Thickness-dependent PEEM topography images reveal that there is a transition from the stripe-domain phase in the thick limit to a single-domain phase in the ultrathin limit, which is dominated by dipolar interaction. Patterning the Fe_3_GeTe_2_ flake into diamond-shaped or rectangular microstructures can greatly enhance the *T*_C_ of Fe_3_GeTe_2_ to room temperature. More interestingly, a spin reorientation from an out-of-plane stripe-domain phase to an in-plane vortex phase occurs at 230 K. Fujita et al. also utilized the XPPEM to study the layer-dependent magnetic domains in 2D ferromagnetic Fe_5_GeTe_2_.^75^ It is found that labyrinth-type domains exist in the bulk sample while fragmented domains are in thinner flakes. Moreover, patterns of the magnetic domain change obviously when the thickness is below five layers. As shown in Figure 4G, the magnetic bubbles and stripes were found in four-layer and trilayer flakes, while a largely isotropic fragmented state was observed in the bilayer. This suggests the reorientation transition driving the magnetic ordering. In research of skyrmions in van der Waals ferromagnet Fe_3_GeTe_2_ on (Co/Pd)_n_ superlattice,^76^ the XPEEM is used to measure the surface magnetization of Fe_3_GeTe_2_. Figure 4H shows the XPEEM images of the FGT magnetic domains at three different thicknesses of the Pd spacer layer. The bright and dark stripes represent the magnetizations in the +*z* and −*z* directions, respectively. It is obvious that dark stripes are gradually broken into bubbles (skyrmions) as the coupling strength increases by decreasing the Pd thickness. It suggests the induced skyrmions are ascribed to the effect of magnetic coupling to the perpendicularly magnetized [Co/Pd]_n_. These results demonstrated that PEEM takes advantage of visually recording the evolution of magnetic domains of 2D magnets at the nanoscale.

#### Scanning tunneling microscopy (STM)

**Figure 5:**
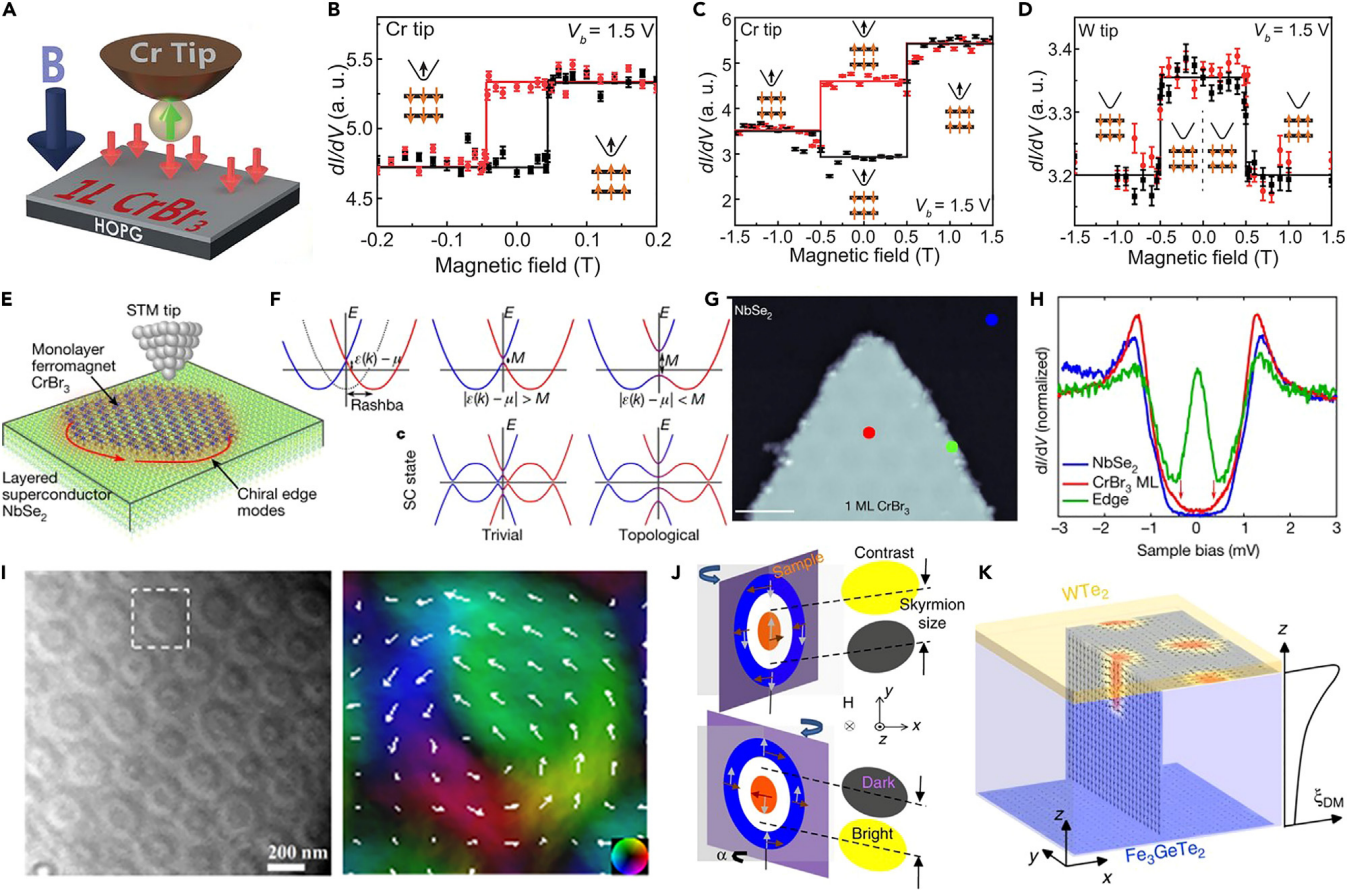
Magnetism detection by STM and LTEM techniques (A) Schematic illustration of STM experimental geometry of 2D CrBr_3_. (B) Spin-polarized tunneling on the H-type stacked bilayer CrBr_3_ as a function of magnetic field with a Cr tip. (C) Spin-polarized tunneling on an R-type stacked bilayer CrBr_3_ with a Cr tip. (D) Spin-polarized tunneling on an R-type stacked bilayer CrBr_3_ with a W tip. (A–D) are reproduced with permission from Chen et al.^45^ Copyright © 2019, American Association for the Advancement of Science. (E) Schematic illustration of STM measurements of 2D CrBr_3_/NbSe_2_ heterostructures. (F) Schematic of the band-structure engineering to realize topological superconductivity. (G) STM image of a monolayer-thick CrBr_3_ island grown on NbSe_2_ (scale bar: 10 nm). (H) Experimental d*I*/d*V* spectroscopy collected at the corresponding point in (G). (E–H) are reproduced with permission from Kezilebieke et al.^78^ Copyright © 2020, Spring Nature. (I) The overfocused Lorentz-TEM images of the skyrmion bubbles taken at 93 K and in zero-field together with the enlarged in-plane magnetization distribution map. Reproduced with permission from Ding et al.^80^ Copyright © 2020, American Physical Society. (J) Schematic diagram of LTEM measurements of Néel-type skyrmions. (K) Simulation results of interfacial Dzyaloshinskii–Moriya interaction in Fe_3_GeTe_2_/WTe_2_ heterostructures. (J and K) are reproduced with permission from Wu et al.^82^ Copyright © 2020, Spring Nature.

Scanning tunneling microscopy (STM) uses the generated tunneling current to obtain the surface information of the sample when the probe is close to the sample surface. It takes a unique advantage in acquiring the topography of surfaces and obtaining the distribution of the electronic density of states. In 2019, Chen et al. used *in situ* spin polarization scanning tunneling microscopy to directly correlate the atomic lattice structure with the observed magnetic structure (Figure 5A), and clarify the association between the stacked structure of bilaterally layered CrBr_3_ and the interlayer coupling.^45^ The bilayer CrBr_3_ is stacked with two different structures: H-type and R-type. In the R-type stacking structure, the top and bottom layers of the bilayer are arranged in the same direction, while in the H-type stacking structure, the top and bottom layers aligned with a 180° rotation. The hysteresis loops of H-type stacked CrBr_3_ bilayers show two platforms (Figure 5B), while R-type stacked CrBr_3_ bilayers exhibit four platforms (Figures 5C and 5D). H-type stacked CrBr_3_ bilayer shows ferromagnetic coupling and the R-type stacked bilayer is antiferromagnetically coupled. STM gives robust experimental support that the interlayer magnetic coupling of 2D magnets is strongly correlated to stacking order. In Fe_5_GeTe_2_, Fe(1) site has two possible positions and plays a major role in magnetic ordering. In order to clarify this crucial issue, Ly et al.^77^ revealed that the regular arrangement of Fe(1) forms a $\sqrt{3}$ ×$\sqrt{3}$ superstructure through STM topography. $\sqrt{3}$ ×$\sqrt{3}$ orderings break the centro-symmetricity and cause the helical magnetism in the Fe_5-x_GeTe_2_. STM provides the understanding of Fe(1) orderings for chiral or helical ordering in the Fe_5−x_GeTe_2_.

In addition to the detection of 2D magnetism, STM enables the direct visualization of many quantum phenomena based on 2D magnetic heterostructures. The one-dimensional Majorana edge modes in vdWs heterostructure constructed by 2D ferromagnet CrBr_3_ with a superconductor NbSe_2_ were revealed by scanning tunneling microscopy and spectroscopy (Figures 5E and 5F).^78^ Figure 5G shows the constant-current STM image of a monolayer CrBr_3_ grown on NbSe_2_. The scanning tunneling spectroscopy measurement was carried out at different locations marked in Figure 5H. The obtained d*I*/d*V* spectroscopy clearly shows a peak localized at *E*_*F*_ together with side features at the edge of CrBr_3_/NbSe_2_, which is a signature of expected Majorana modes. To conclude, STM provides ultrahigh resolution surface topography and electronic states of materials, which correlates magnetic order with the electronic structure in 2D systems.

#### Lorentz transmission electron microscopy (L-TEM)

The principle of Lorentz electron microscope is to use the electron beam to pass through the sample. When the electron beam passes through the film, it is deflected by the Lorentz force of the intrinsic magnetic field in the sample. The deflection of the electron beam on the deflector will produce focusing and under-focusing phenomena, which are shown as black-and-white areas.^79^ Topologically vortex-like magnetic spin textures including the (anti)skyrmion, biskyrmion, (anti)meron, and bobber are the research hotspots. L-TEM takes a unique advantage in detecting this particular kind of magnetic signals. A recent work employed *in situ* L-TEM to record the formation of Bloch-type magnetic skyrmions in single crystals of 2D vdWs Fe_3_GeTe_2_ (FGT).^80^ When applied the magnetic field increased from 0 to 680 Oe, the magnetic stripe domains gradually transformed into magnetic skyrmion bubbles in FGT thin plates. More importantly, the magnetic skyrmion bubbles can keep stable after removing the magnetic field as shown in Figure 5I. The theoretical simulations of spin textures matched well with the experimental results. At the same time, Bloch-type skyrmionic bubbles in exfoliated 2D vdWs Cr_2_Ge_2_Te_6_ are also directly captured by L-TEM.^81^ In addition to single vdWs ferromagnets, the skyrmion can also be realized at the interface of the magnetic heterostructure.^82^ In contrast to Bloch-type skyrmions, the observations of Néel-type skyrmions require titling angles between the sample and magnetic fields (Figure 5J). Wu et al. used the L-TEM to directly image the Néel-type skyrmion lattice in WTe_2_/FGT heterostructure, which is attributed to the Dzyaloshinskii–Moriya interaction (DMI) caused by the strong interfacial coupling between the 2D ferromagnet and heavy metal (Figure 5K). Yang et al. also demonstrated the magnetic interface coupling interaction is responsible for stable Néel-type skyrmion in Fe_3_GeTe_2_ on (Co/Pd)_*n*_ superlattice rather than the external magnetic field.^76^ A new type of topological magnetic excitation, known as domain wall meron chains, was observed in the 2D vdW ferromagnetic material Fe_5-x_GeTe_2_ by L-TEM.^83^ These chains emerge through the evolution of 180° magnetic domain walls. This topological excitation is induced by a spin reorientation occurring as the magnetic anisotropy transitions from the *c*-axis to the *ab*-plane upon decreasing temperature, which is originally attributed to the varying ordering of Fe(1) atoms and confirmed by the above STM observation. Later, Casas et al.^84^ through the L-TEM to reveal the coexistence of merons with skyrmions in Fe_5-x_GeTe_2_, which originated from the distinct regions of in-plane and out-of-plane magnetic anisotropy. The L-TEM technique plays a key role in the detection of topological spin textures, offering a direct image of spin texture evolution with the magnetic field or sample tilting angles.

#### Scanning single-spin magnetometry

**Figure 6:**
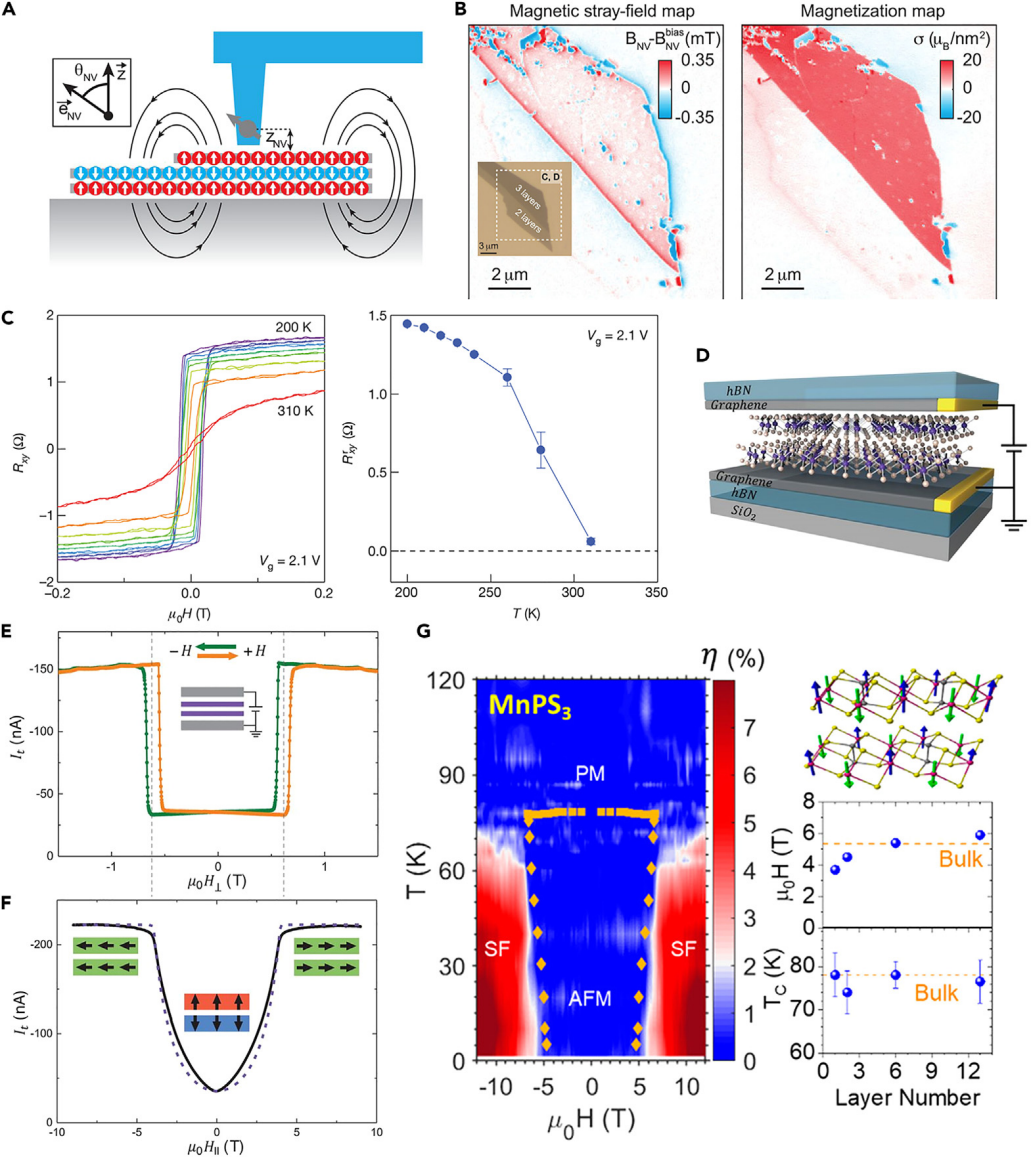
The detection of 2D magnetism via the scanning single-spin magnetometry and electrical techniques (A) Schematic diagram of the scanning single-spin magnetometry measurement on 2D CrI_3_. (B) Magnetic field map of B_NV_ and magnetization distribution across the sample (inset showing the optical image). (A and B) are reproduced with permission from Thiel et al.^88^ Copyright © 2019, American Association for the Advancement of Science. (C) Temperature of *R*_xy_ for a four-layer FGT flake under a gate voltage of *V*_g_ = 2.1 V. Reproduced with permission from Deng et al.^43^ Copyright © 2018, Spring Nature. (D) Schematic of 2D magnetic tunnel junction with bilayer CrI_3_ as the tunneling barrier. (E) Tunneling current as a function of out-of-plane magnetic field. (F) Tunneling current as a function of in-plane magnetic field. (D, E, and F) are reproduced with permission from Song et al.^98^ Copyright © 2018, American Association for the Advancement of Science. (G) Spin-flop transition in thick MnPS_3_ multilayers probed by tunneling magnetoresistance. Reproduced with permission from Long et al.^99^ Copyright © 2020, American Chemical Society.

Quantum magnetometers based on a single Nitrogen-Vacancy (NV) center in diamond have recently received significant attention in magnetic imaging.^85^ The single nitrogen-vacancy (NV) defect in diamond was employed as a noninvasive and nanoscale quantum sensor to image weak static and dynamic magnetic stray fields, which takes a unique advantage in providing magnetic information with high sensitivity and high spatial resolution.^86^^,^^87^ It is highly recognized that NV magnetometry was recently employed to investigate the magnetism in 2D vdWs magnets, including CrI_3_,^88^ MnBi_4_Te_7_,^89^ CrBr_3_,^90^ VI_3_,^91^ CrTe_2_,^92^ and moiré superlattice of twisted stacking CrI_3_.^55^ Currently, application of the NV magnetometry in studying 2D magnets can divide into two approaches, including scanning NV microscopy and wide-field NV microscopy.^87^ The first approach was first demonstrated by quantitatively analyzing the magnetism of CrI_3_. The determination of the stray magnetic field of 2D CrI_3_ is obtained by detecting the spin states of NV defect in diamond. The method of reading the spin states with light excitation is often called optical detected magnetic resonance (ODMR). Figure 6A shows the schematic of the scanning single-spin magnetometry technique employed on the trilayer and bilayer CrI_3_.^88^ Strong stray fields emerge from the edges of the trilayer flake as shown in Figure 6B. By reverse propagation, the magnetization map was presented on the right of Figure 6B. It clearly shows a homogeneous magnetization in trilayer CrI_3_ and zero magnetization in bilayer CrI_3_, consistent with the previous observation of antiferromagnetic interlayer exchange coupling. This was further supported by mapping the magnetization of different CrI_3_ samples with even and odd numbers of layers. More importantly, magnetic domains in the nine-layer CrI_3_ flake were directly imaged, where the magnetization is discretized in integer multiples of the monolayer magnetization. This is ascribed to the spatial variation of the stacking order. McLaughlin et al. also employed scanning NV magnetometry to study the domain wall nucleation and propagation features during the spin-flip transition of MnBi_4_Te_7_ flakes,^89^ which can provide an understanding of magnetic domain dynamics for underlying spin transport physics. The emergence of the FM and AFM magnetic domains in twisted bilayer CrI_3_ was observed, which is strongly related to the spatial variation of local stacking-dependent interlayer coupling as demonstrated by the scanning NV magnetometry measurement.^55^ The representative application of the second approach NV wide-field microscopy is to explore the mechanism of switching processes in ultrathin VI_3_.^91^ By imaging the domain reversal and initial magnetization, it is found that the anisotropy-limited domain wall nucleation processes govern the coercivity of ultrathin VI_3_. NV magnetometry is still an emerging technique, but the demonstrations of using NV magnetometry highlight it as an ideal quantum sensing platform for 2D magnets or related 2D magnetic heterostructures in the future.

### Electrical techniques

#### Anomalous Hall effect (AHE)

Generally, the Hall resistance of a ferromagnetic material consists of two contributions: an ordinary Hall effect (OHE) term and the anomalous Hall effect (AHE). The first part is due to the Lorentz force acting on the charge carriers and the second term is proportional to the magnetization. Based on this theory, the overall Hall effect of magnetic materials can be expressed by the following formula.

(Equation 1) $$R_{xy}\left(H\right)=R_{0}B_{Z}+R_{S}M_{Z}$$

where *R*_0_ is the ordinary Hall coefficient determined from a linear fitting of *R*_*xy*_ at high magnetic fields, *R*_s_ is the anomalous Hall coefficient of the ferromagnetic component. *B* and *M* are the out-of-plane components of the magnetic field and the magnetization of the magnetic material itself, respectively. Thus, the AHE-based measurements are an effective technique to characterize the ferromagnetism of vdWs ferromagnets. According to Equation 1, due to the much weaker OHE, *R*_*xy*_ is proportional to *M*, thus magnetic properties such as coercive field, magnetization reversal, and saturation moment can be directly obtained by measuring *R*_*xy*_.^34^ The first demonstration of robust ferromagnetic order down to the monolayer in the popular ferromagnetic metal FGT was through AHE measurement.^43^ The strong dimensionality effect on the ferromagnetism in FGT was obtained by analyzing the anomalous Hall resistance data. Moreover, as shown in Figure 6C, AHE visually shows that FGT can maintain room temperature ferromagnetism under an ionic gate. Later, AHE is widely employed to investigate the exchange bias effect of vdWs ferromagnetic/antiferromagnetic heterostructures, such as CrOCl/FGT,^56^ FePS_3_/Fe_5_GeTe_2_,^93^ molecule/FGT,^94^ and FGT/MnPX_3_ (X = S and Se).^95^ In the CrCl_3_/FGT heterostructure,^96^ the *R*_*xy*_ loops shift to the negative *H* direction, indicating a clear exchange bias effect. The key parameter exchange fields were obtained from the data of *R*_*xy*_ according to *H*_E_ = (*H*_CR_ + *H*_CL_)/2, where the *H*_CR_ and *H*_CL_ represent coercivities at positive and negative fields, respectively. For the ferromagnets with metallic conductive properties, AHE is an effective way to detect the ferromagnetism and study the related magnet-transport properties.

#### Tunneling transport

Electron tunneling is strongly dependent on the spin state, resulting in tunneling magnetoresistance (TMR). Thus, the tunneling transport can reflect the magnetic ordering or magnetic state of materials.^97^ When it comes to ferromagnetic materials, when the magnetization of two ferromagnetic layers is arranged in parallel, the TMR can reach a maximum value. On the contrary, the tunneling resistance will have a minimum value if FM layers are antiparallel aligned. For ferromagnetic metals, insulating material (e.g., *h*-BN) is sandwiched by two ferromagnetic metals to construct a magnetic tunneling junction (MTJ). The FGT/BN/FGT heterostructure was designed as MTJ, where the top and bottom FGT has different thickness. The magnetic field corresponding to the tunneling resistance increases abruptly and subsequently decreases, which is consistent with the reversal of the magnetization of FM layers. Therefore, the magnetization information can be deduced from the TMR. While in the case of insulating ferromagnets, they always served as tunneling layers to explore electron tunneling. As shown in Figure 6D, the atomically thin chromium triiodide (CrI_3_) acts as a spin-filter tunnel barrier, and the conductive graphene is used as electrodes.^98^ The tunneling current shows a lower plateau at low fields. When the magnetic field increases to a critical point, the tunneling current jumps to a higher plateau (Figure 6E). The tunneling current as a function of the magnetic field exactly corresponds to the conversion of the layered antiferromagnetic ground state (↑↓ or ↓↑) to fully spin-polarized states (↑↑ and ↓↓). The spin-conserving tunneling current of electrons is indicative of the spin state of two FM layers. Moreover, the magnetic anisotropy can also be checked by tunneling current measurements. As shown in Figure 6F, the tunneling current increases as the in-plane magnetic field increases from 0 to 4 T and then saturates at high fields. The critical magnetic field is simulated to be 3.8 T, which is much larger than the situation of out-of-plane critical magnetic field (0.6 T). This indicates a large out-of-plane magnetic anisotropy of bilayer CrI_3_.

To clarify the persistence of magnetism in 2D MnPS_3_, temperature-dependent tunneling magnetoresistance measurement was employed to obtain its magnetic phase diagram, where the MnPS_3_ was used as a tunnel barrier.^99^ Figure 6G shows the color plot of the tunneling magnetoresistance of 6-layer MnPS_3_ as a function of temperature and out-of-plane magnetic field. It is clear that the tunneling magnetoresistance can track the magnetic phase boundary between the antiferromagnetic state at low field and the spin-flop at high field. The Temperature-dependent characteristic fields *H*_1_ obtained from magnetoresistance coincide with the spin-flop field *H*_sf_. The change in tunneling magnetoresistance is attributed to differences in the height of the tunnel barrier in the antiferromagnetic and in spin-flop states. And the similar phenomenon was observed in mono- and bilayer MnPS_3_. This work experimentally establishes quantitative information on the magnetic states in antiferromagnetic systems. In a word, tunneling transport studies have been proven to be an effective and operable method to characterize and explore the magnetic state of 2D materials.

## Modulation of 2D magnetism

Due to the weak interlayer van der Waals forces, the magnetic state in 2D magnets is easily regulated by a variety of external stimuli (magnetic field, electric field, doping, stack method, strain, pressures, and interface, etc.). Herein, we will summarize the modulation strategies and corresponding mechanisms that have been developed so far.

### Electrical modulation

**Figure 7:**
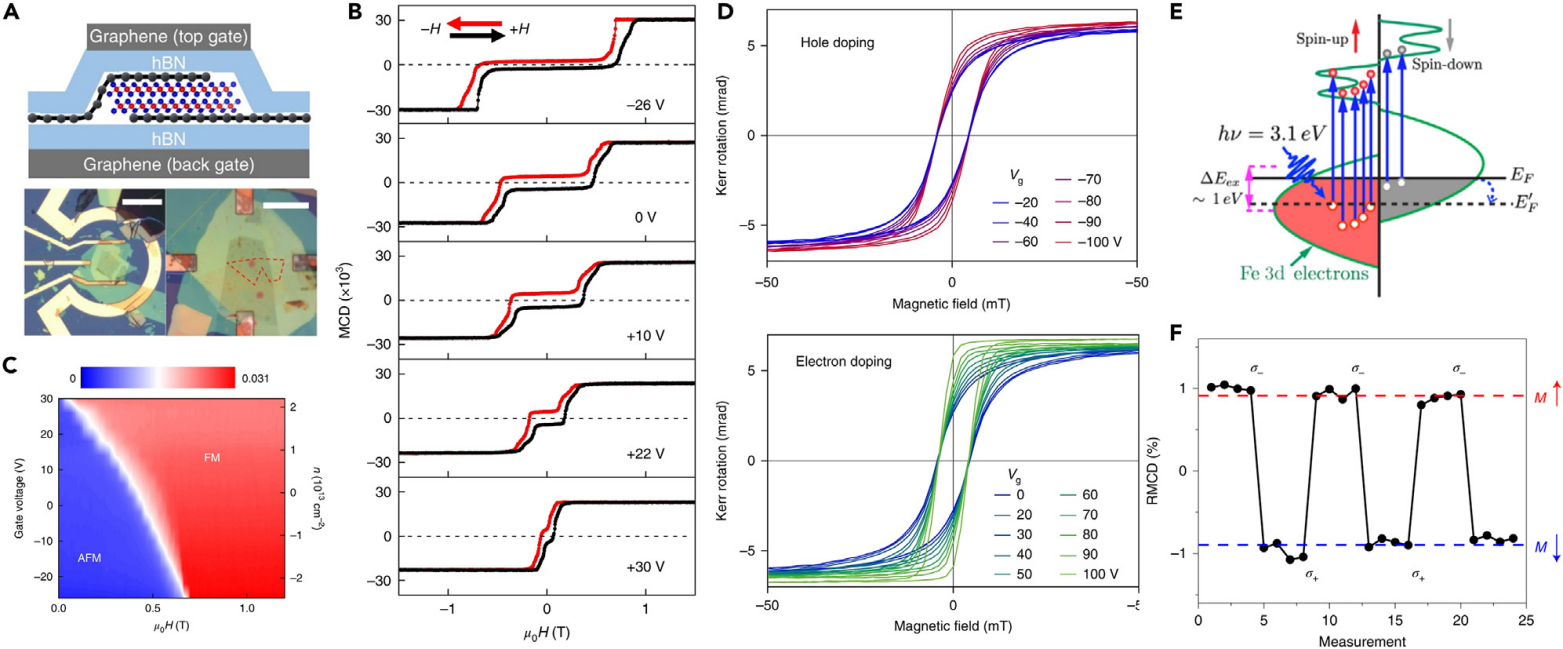
Modulation of 2D magnetism via electrostatic gating and optical fields (A) A schematic side view and the optical image of a dual-gate bilayer CrI_3_ field-effect device (scale bars, 50 μm for left panel and 20 μm for right panel). (B) MCD measurements of dual-gate bilayer CrI_3_ field-effect device as a function of magnetic fields at representative gate voltages. (C) Doping density–magnetic field phase diagram. (A–C) are reproduced with permission from Jiang et al.^102^ Copyright © 2018, Spring Nature. (D) Kerr measurement of thin Cr_2_Ge_2_Te_6_ sample under the positive and negative gate voltages. Reproduced with permission from Wang et al.^104^ Copyright © 2018, Spring Nature. (E) Schematic diagram of the laser-excited DOS in few-layered FGT. Reproduced with permission from Liu et al.^108^ Copyright © 2020, American Physical Society. (F) Repeated deterministic switching of magnetization states by circularly polarized pulsed irradiation. Reproduced with permission from Zhang et al.^109^ Copyright © 2022, Spring Nature.

By directly applying an electrostatic field or ionic-liquid gate perpendicular to the material, the external electric field can be introduced into the interlayer direction of the 2D magnetic material. The applied electric field or charge doping can greatly adjust the carrier concentration, orbital occupancy number, and electronic structure of materials, and then affect the magnetic ground state, magnetic exchange interaction, and magnetic anisotropy of materials, which is a class of effective ways to control the magnetic properties of 2D materials.^100^^,^^101^ Jiang et al. successfully modulated the magnetic properties of monolayer and bilayer CrI_3_ by electrostatic doping in CrI_3_-graphene vertical heterostructures.^102^ In this heterostructure, CrI_3_ was encapsulated using BN and attached to a graphene electrode, sandwiched between two conductive materials (Figure 7A). The dual gate voltage device can effectively distinguish the effect of the applied vertical electric field from the accumulated charge carriers experimentally. It is found that in monolayer CrI_3_, the electrostatic doping effect changes the saturation magnetization, coercivity, and Curie temperature, which is enhanced/weakened with the hole/electron doping magnetic order. Figure 7B illustrates the impact of electrostatic doping on bilayer CrI_3_. As the electron doping density *n* increase, the spin-flip transition field H_*sf*_ exhibits a drastic decrease. The interlayer exchange coupling is significantly modified by electron doping. The interlayer exchange constant *J*_⊥_ can be expressed as: 2 *J*_⊥_ = $\mu_{0}M_{s}\left(M_{sf}-M_{s}/2t\right)$ , where *t* is the interlayer separation, ∼0.7 nm. Through the measured H_sf_ and M_s_, we can get the dependence of *J*_⊥_ with the doping level. *J*_⊥_ exhibits a monotonic decrease and undergoes a change in sign with electron doping. In the bilayer CrI_3_, electron doping above ∼2.5×10^13^ cm^−2^ leads to a conversion from the antiferromagnetic ground state to the ferromagnetic ground state without a magnetic field (Figure 7C). Such a phenomenon is attributed to the magnetoelectric coupling effect controlled by the spin order. The magnetoelectric coupling coefficient reaches 110 ps/m, which exceeds most single-phase magnetoelectric coupling materials. This effect can be used to realize the robust switching of magnetization in bilayer CrI_3_ through a small gate voltage. The switching between antiferromagnetic and ferromagnetic states controlled by voltage in bilayer CrI_3_ was also demonstrated by another research group.^103^

Wang et al. also successfully realized bipolar all-electric control of charge and spin in a 2D ferromagnetic semiconductor Cr_2_Ge_2_Te_6_ thin layer via electrostatic gating.^104^ As shown in Figure 7D, the hysteresis loop of 2D Cr_2_Ge_2_Te_6_ can be effectively tuned by electron or hole doping as illustrated by the Kerr measurements. Through the hole (electron) doping, the Fermi level of Cr_2_Ge_2_Te_6_ will be shifted into the valence (conduction) band by depleting (filling) Te-p (Cr-d) orbitals. The spin minority and spin majority states contribute to the bands near the valence band maximum (VBM) and conduction band minimum (CBM), respectively. The calculation results reveal net magnetic moments both increase with the hole and electron doping. This suggests the feasibility of spin field-effect devices based on 2D van der Waals ferromagnets. Compared with electrostatic doping, ionic liquid forms a dielectric layer of nanometer thickness at the solid-liquid interface, which is a more effective means to greatly regulate the carrier concentration of materials. Verzhbitskiy et al. used the ionic liquid (DEME-TFSI) as a gate to modulate the magnetic properties of Cr_2_Ge_2_Te_6_.^105^ Based on an electric double-layer transistor geometry, a high doping level is achieved in Cr_2_Ge_2_Te_6_, in which the carrier concentration reaches 4 × 10^14^ cm^−2^. Under such large carrier densities, the double exchange mechanism prevails over the superexchange mechanism, and thus further stabilizes the ferromagnetic order. The Curie temperature of Cr_2_Ge_2_Te_6_ increases to 200 K from 61 K, and the easy magnetization axis turns from out-of-plane to in-plane. Traditional ionic liquids are inconvenient in practical applications because of their liquid nature. Tan et al.^106^ utilized a solid protonic gate to realize a high electron doping concentration of above 10^21^ cm^−3^ in ferromagnet Fe_5_GeTe_2_, which exceeds that of the widely used double-layer transistors. More interestingly, a transition from ferromagnetism to antiferromagnetism (AFM) occurred at *V*_g_ = −5 V. Electrical control of magnetism would be highly desirable in the development of magneto-electronics and spintronics from fundamental and technological viewpoints.

### Optical field

The optical field provides a new way to control the magnetism of 2D materials. The light could trigger heating and photo-doping and thus can cause a modification of the exchange interaction, which was demonstrated in traditional magnetic materials.^107^ Recently, Liu et al.^108^ reported that a femtosecond laser pulse can strongly modulate the ferromagnetism in few-layered Fe_3_GeTe_2_ (FGT). It is observed that the saturation magnetization gets enhancement and coercivity decreases monotonically when the laser intensity increases. The laser pulse also drives the realization of room temperature ferromagnetism in FGT. It is found that the photo-generated carrier doping effect changes the electronic structure, which plays an important role in photo-regulated magnetism (Figure 7E). The photo-generated carrier near the Fermi level results in a redistributed electronic state and thus an enhanced density of state (DOS). The ferromagnetic order was strengthened by the enhanced DOS, manifesting improved *T*c and decreased magnetic anisotropy. When the Fermi level shifts, the band structure window, which is mainly influenced by spin-orbital coupling, moves further away from the Fermi level and leads to a suppression of uniaxial anisotropy. This work provides new experimental evidence to understand the magneto-optical physical mechanism of 2D ferromagnetism. Beyond that, Zhang et al. demonstrated selective and deterministic magnetization switching in CrI_3_ utilizing a circularly polarized pulsed laser.^109^ As shown in Figure 7F, we can clearly observe the magnetization in CrI_3_ flakes is selectively switching after the circularly polarized pulsed laser ($\sigma_{-}$ and $\sigma_{+}$) illumination. Interestingly, the switching threshold behavior is strongly dependent on the energy and polarization of the pulsed laser. The switching mechanism is ascribed to the spin angular momentum transfer between the excited carriers and the magnetic moment. All in all, the light-controlled magnetism provides a new platform to understand the strong light-matter interaction of 2D vdWs magnets and develop advanced spin optoelectronic devices.

### Mechanical methods: Pressure and strain

**Figure 8:**
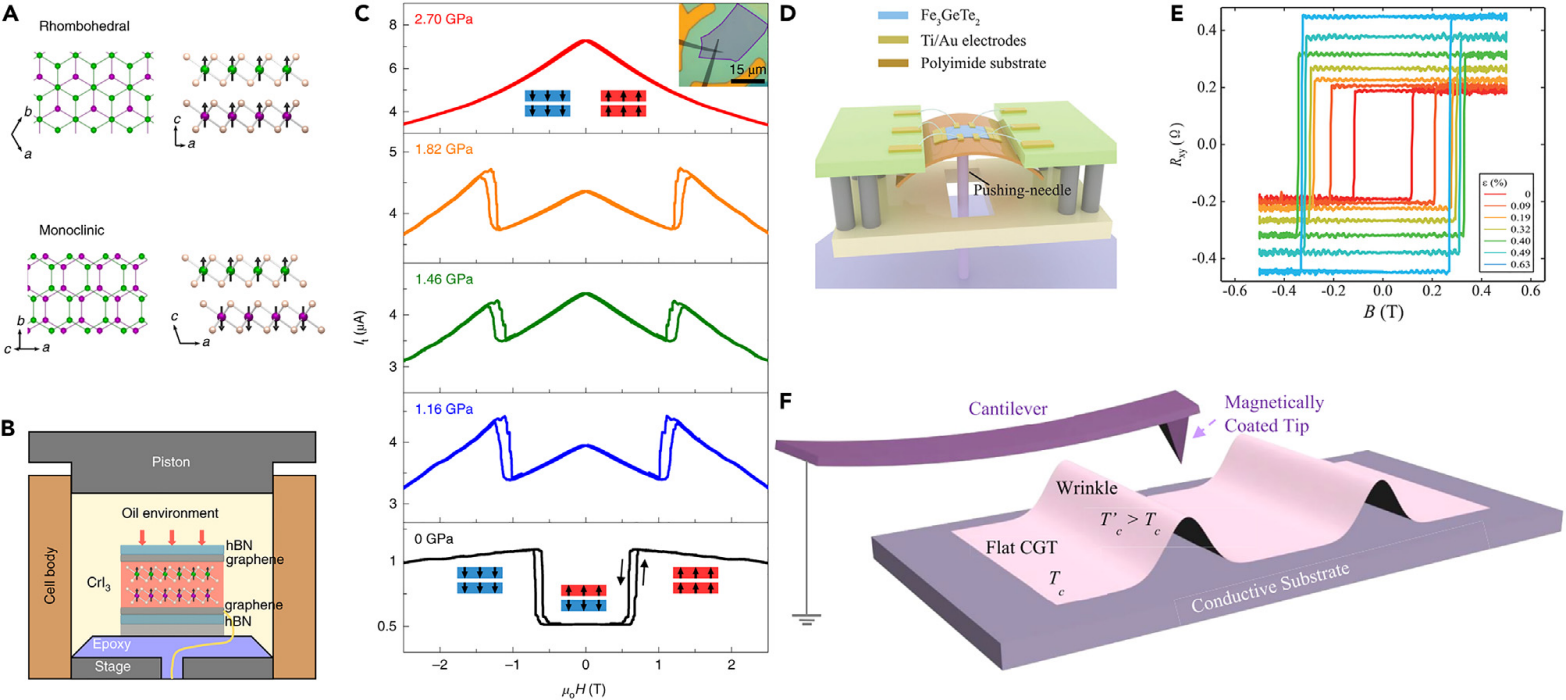
Manipulation of 2D magnetism via stress and strain (A) Schematic of rhombohedral stacking and monoclinic stacking of CrI_3_. (B) Schematic of high-pressure experimental set-up of bilayer CrI_3_. (C) Tunneling current versus magnetic field of bilayer CrI_3_ device at a series of pressures. (A–C) are reproduced with permission from Song et al.^111^ Copyright © 2019, Spring Nature. (D) Schematic diagram of FGT device in the strain experimental set-up. (E) The *R*_xy_ versus *B* curves of FGT under a series of strains from 0 to 0.63%. Figures A and B are reproduced with permission from Wang et al.^112^ Copyright © 2020, Wiley-VCH. (F) Schematic image of wrinkles in layered Cr_2_Ge_2_Te_6_ and its MFM measurements. Reproduced with permission from O’Neill et al.^113^ Copyright © 2023, American Chemical Society.

For 2D magnets, the interlayer exchange coupling strongly depends on the layer spacing. And the stacked structure can even change the sign of the interlayer magnetic exchange, thus dramatically changing the magnetic ground state. Mechanical regulation is mainly divided into strain regulation and pressure regulation. The principle of this regulation is to change the cell parameters and lattice structure of 2D materials by external forces. Lattice distortion leads to the change of magnetic anisotropy energy and spin exchange coupling. Li et al. applied hydrostatic pressures of up to 2 GPa to change the stacking order in the van der Waals magnetic insulator CrI_3_.^110^ Irreversible interlayer antiferromagnetic to ferromagnetic transitions in atomically thin CrI_3_ were observed by magnetic circular dichroism and electron tunneling measurements. Raman characterization shows that the effect is accompanied by a change in the stacking order of the monoclinic rhomboid phase. The interlayer antiferromagnetic coupling energy can be adjusted by nearly 100% by pressure prior to structural change. The correlation between the magnetic ground state and stacking order observed in this experiment is in good agreement with first-principles calculations. Meanwhile, Song et al. also reported pressure-regulated magnetism in 2D magnet CrI_3_,^111^ as shown in Figures 8A–8C. In fact, it is difficult to apply pressure on the atomically thin layers. The CrI_3_ device is held in a piston pressure cell (Figure 8B). A force applied to the piston generates pressure on the CrI_3_ device through the oil environment. It is found that hydrostatic pressure can more than double the magnetic coupling between layers. In the double layer CrI_3_, the pressure will change the antiferromagnetic phase to the ferromagnetic phase (Figure 8C). In three layers of CrI_3_, the pressure can generate three phases of coexisting magnetic domains, one ferromagnetic phase, and two antiferromagnetic phases. The observed shift in magnetic order can be explained by a change in stacking order.

In addition to pressure control, tensile or compressive strain can directly affect the coupling strength and mode between magnetic atoms and then change the 2D magnetism. Wang et al.^112^ have successfully fabricated Fe_3_GeTe_2_ (FGT) devices on polyimide (PI) flexible substrates (Figure 8D) and studied Hall resistance *R*_*xy*_ under different strain intensities (Figure 8E). It was found that the hysteresis window would gradually increase with the increase of strain. When the strain is increased to 0.32%, the coercive field increases by more than 150%, which shows that the strain has a significant regulating effect on magnetism. With the strain increasing from 0% to 0.65%, the *T*_C_ of Fe_3_GeTe_2_ film changes from 180 K to 210 K. First-principles calculations show that the observed strain-regulated behavior is due to the change of magnetic anisotropy energy (MAE). As strain is applied within the range from 0.0% to 0.6% in the FGT sample, the orbital portion of the magnetic moment is enlarged during the lattice expansion. Then the enhanced spin-orbit coupling (SOC) effect determined an increase in MAE. Very recently, the highly strained and wrinkled Cr_2_Ge_2_Te_6_ sheets show significant improvement in Curie temperature^113^ as shown in Figure 8F. It is worth mentioning that strained wrinkles were obtained by mechanically bending the flexible substrates. The compressive strain on the wrinkled Cr_2_Ge_2_Te_6_ sheets depends on the extent of the buckling. Here, the application of strain changes the super-exchange interaction and thus the increase of the magnetic anisotropy energy.

### Chemical doping

**Figure 9:**
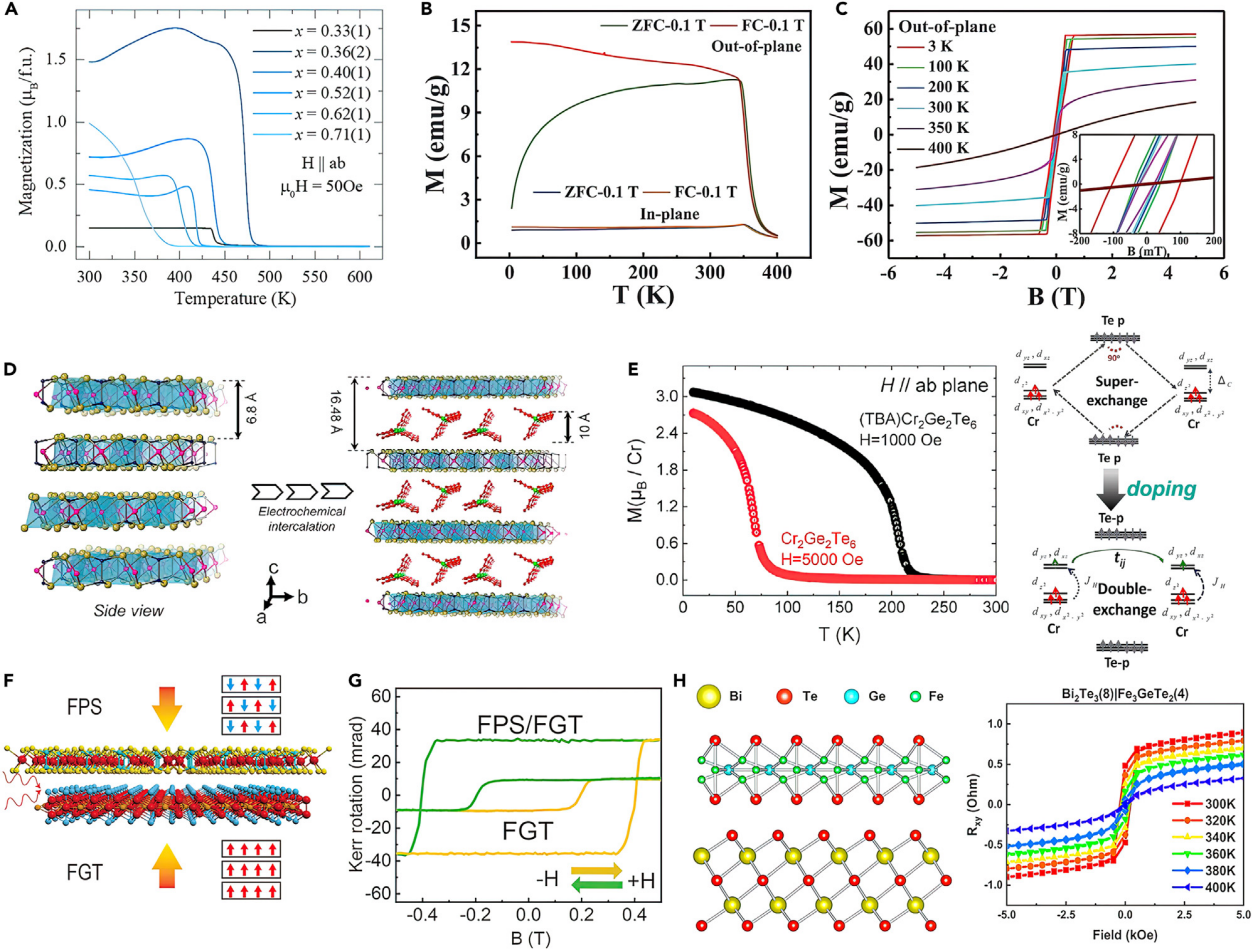
Modulation of 2D magnetism via chemical doping and interface engineering (A) Temperature-dependent magnetization of Ni-Fe_5_GeTe_2_ at selected Ni doping level *x*. Reproduced with permission from Chen et al.^120^ Copyright © 2022, American Physical Society. (B) Temperature-dependent spontaneous magnetization curves of Fe_3_GaTe_2_ bulk crystals (B = 0.1 T, out-of-plane). (C) M-H curves of Fe_3_GaTe_2_ bulk crystals at varying temperatures with magnetic fields along the out-of-plane. (B and C) are reproduced with permission from Zhang et al.^36^ Copyright © 2022, Spring Nature. (D) The crystal structures of Cr_2_Ge_2_Te_6_ and (TBA)Cr_2_Ge_2_Te_6_. (E) Temperature-dependent magnetic susceptibility (M-T) and the schematic diagrams of magnetic interaction of pristine Cr_2_Ge_2_Te_6_ and (TBA)Cr_2_Ge_2_Te_6_, respectively. (D and E) are reproduced with permission from Wang et al.^128^ Copyright © 2019, American Chemical Society. (F) Schematic diagrams of FePS_3_/FGT heterostructures. (G) Typical MOKE hysteresis loop for an FGT flake and an FPS/FGT heterostructure. (F and G) are reproduced with permission from Zhang et al.^130^ Copyright © 2020, Wiley-VCH. (H) Schematic diagrams of Bi_2_Te_3_/FGT heterostructures. Reproduced with permission from Wang et al.^134^ Copyright © 2020, American Chemical Society.

Chemical doping, such as elemental substitution or increasing the stoichiometric ratio of the magnetic element, is a traditional way to modulate intrinsic magnetism, which has been widely used for traditional magnetic materials. In the case of Fe_3-*x*_GeTe_2_, the iron content deeply affects its magnetic properties. Curie temperature, saturation magnetization, and coercive field are decreased when Fe content is insufficient.^114^ Moreover, when the iron content is increased to 5, the Curie temperature can be greatly increased up to room temperature (270–310 K).^115^ The magnetic ground state of Fe_5-*x*_GeTe_2_ becomes more complex compared to that of FGT. Zhang et al.^116^ claimed that Fe_5-*x*_GeTe_2_ undergoes the two magnetic phase transition as temperature decreases, including the ferromagnet to ferrimagnet at 275 K and glassy clusters below 110 K. While another work revealed Fe_5-*x*_GeTe_2_ transform from paramagnetic to ferromagnetic state at 265 K and then evolves to a ferrimagnetic state at 100 K.^117^ Meanwhile, elemental substitution also greatly affects the magnetism of Fe_5_GeTe_2_. By cobalt (Co) substitution of Fe_5-*x*_GeTe_2_, tunable magnetic properties were obtained in van der Waals crystals (Fe_1*-x*_Co_*x*_)_5_GeTe_2_.^118^ When *x* = 0.2, the axis of easy magnetization turns from out-of-plane to in-plane, and the Curie temperature increase from 276 K to 337 K. While 44% of Co doping induced an AFM ground state with *T*_N_ = 335 K. More strikingly, room temperature skyrmion lattice was observed in 50% Co–doped Fe_5_GeTe_2_.^119^ In the case of nickel (Ni) substitution in (Fe_*1-x*_Ni_*x*_)_5_GeTe_2_, the highest *T*_C_ (478 K) is achieved when the Ni doping level of *x* is 0.36^120^ as shown in Figure 9A. In contrast, Ni doping in Fe_3_GeTe_2_ will dilute the ferromagnetism, causing the decrease of Curie temperature and effective magnetic moments.^121^

In addition to the substitution of magnetic elements of the FGT series, doping of non-magnetic elements also could strongly affect the magnetic properties of materials. May et al. study the doping effect through chemical substitution at the nonmagnetic (Ge) of Fe_5_Ge_1−y_As_y_Te_2_.^122^ It is found that the ferromagnetic Curie temperature (*T*_C_) and saturation magnetization could decrease with larger arsenic concentrations. While a very small amount of doping (2.5% and 5%) could improve the transition temperature of Fe_5_GeTe_2_. Yang et al.^123^ reported that the Curie temperature of FGT can be greatly enhanced by almost 100% by implanting Ga into Fe_3_GeTe_2_. Moreover, the Ga doping induced the magnetic easy axis of FGT to turn to in-plane from out-of-plane at high temperature. Very recently, the Ge element in Fe_3_GeTe_2_ was completely replaced by the Ga element as a robust ferromagnet.^36^ Fe_3_GaTe_2_ has been reported with above-room-temperature Curie temperature (*T*c, ∼350–380 K), high saturation magnetization (40.11 emu/g), and strong PMA as shown in Figures 9B and 9C. Apart from the Fe_*x*_GeTe_2_, elemental substitution also plays a role in tuning the magnetism of other vdWs magnets. Theoretical calculation shows by substituting the Cr sites with W in CrI_3_ or CrGeTe_3_,^124^ the Curie temperatures of CrWI_6_ and CrWGe_2_Te_6_ monolayers are enhanced by 3∼5 times. Abramchuk et al. experimentally found the magnetic transition temperature of CrCl_3_ can be tuned linearly with the content of Br.^125^ Chemical doping has been proven to be a powerful and robust way to achieve tunable magnetic properties of magnets.

### Intercalation

2D materials are bonded by van der Waals forces. Atoms, ions, and molecules can intercalate into the van der Waals gap and thus change the interlayer spacing or bond strength between layers, which then influences the magnetic exchange interaction of 2D magnetic materials. Huang et al. investigated the Li-ion intercalation into the FGT bilayers based on the first-principles calculations.^126^ The interlayer magnetic coupling energy can be defined as ΔE= (E_AFM_-E_FM_), where E_AFM_ and E_FM_ represent the energies of Li_*x*_-FGT bilayer model in interlayer AFM and FM spin configurations. When at a relatively low intercalation, ΔE increases, and the interlayer ferromagnetic coupling can be enhanced. While the over-intercalation generates a reduced interlayer FM coupling. The calculation results provide a reference for the experiment to select the appropriate intercalation concentration. When intercalating the H atoms into 2D MnPSe_3_ bilayers, H atoms will be bonded to one of the MnPSe_3_ layers.^127^ Calculations results reveal that the hydrogen functionalization breaks the centrosymmetry of bilayer MnPSe_3_ and thus leads to an out-of-plane ferroelectric polarization. Moreover, the H-functionalized MnPSe_3_ transfers from the AFM semiconductor to FM half-metal.

Intercalating various organic cations into the van der Waals gaps can avoid the introduction of defects and impurity phases. Wang et al. employed the electrochemical intercalation of organic molecules to significantly increase the ferromagnetic order temperature of Cr_2_Ge_2_Te_6_.^128^ As shown in Figure 9D, the interlayer spacing of (TBA)Cr_2_Ge_2_Te_6_ is greatly enhanced after intercalating with TBA^+^ cations. Interestingly, (TBA)Cr_2_Ge_2_Te_6_ shows metallic behavior, high Curie temperature up to 208 K (Figure 9E), and easy magnetization direction switching when compared to semiconductor Cr_2_Ge_2_Te_6_. The theoretical calculation revealed that the transition of magnetic exchange interaction is responsible for the huge increase in Curie temperature. A super-exchange interaction in pristine Cr_2_Ge_2_Te_6_ switched to a strong double-exchange interaction in (TBA)Cr_2_Ge_2_Te_6_. After intercalation, doublet d_*xz*_/d_*yz*_ orbits of Cr contribute an effective nonzero orbital moment along the *ab*-plane. Considering the spin-orbit coupling, the magnetic easy-axis of (TBA)Cr_2_Ge_2_Te_6_ switches to the *ab*-plane from the *c*-axis. Another work explores the tetraheptylammonium (THA^+^) into the vdWs gaps of antiferromagnetic insulator NiPS_3_.^129^ The magnetic order of NiPS_3_ transforms from antiferromagnetism to ferrimagnetism and then antiferromagnetism with the increasing of electron concentration. Such variation is ascribed to competition between Stoner exchange and super-exchange.

### Interface engineering

Through van der Waals interaction, 2D magnetic layers can stack on the surface of other 2D magnetic or non-magnetic materials, leading to the modulation of magnetic properties via interface engineering. The degrees of freedom, such as material types and stacking modes, are fully utilized to design various van der Waals interfaces. Various interface coupling mechanisms are possibly generalized including the charge transfer, dielectric screening, super-exchange interaction, band renormalization, and SOC proximity effect.

By coupling with an antiferromagnetic FePS_3_ (Figure 9F), the key parameters of FGT get substantive enhancement.^130^ The *T*c value is greatly improved by ≈ 30 K compared to that of a single FGT. The coercive field of FGT/FePS_3_ is nearly doubled (Figure 9G). The proximity coupling effect of the antiferromagnet/ferromagnet interface causes a change in the spin texture of FGT. Similar demonstrations of enhanced *T*_C_ in other AFM/FM heterostructures such as FGT/CrSb^131^ and NiO/CGT^132^ support the explanation of the proximity coupling effect. Beyond the improvement of *T*_C_ and coercive fields, the exchange bias effect is usually generated in the FM/AFM heterostructure, which has been demonstrated in FGT/MnPX_3_ (X = S and Se),^95^ FGT/CrOCl,^56^ FGT/CrCl_3_,^96^ molecule/FGT.^94^ Wang et al. found coercivity is suppressed in FM/FM heterostructure FGT/Cr_2_Ge_2_Te_6_.^133^ It is shown that charge transfer occurs from metallic Fe_3_GeTe_2_ to semiconducting Cr_2_Ge_2_Te_6_, which leads to a decrease in the local electron density of the interfacial Fe_3_GeTe_2_ layer. Thus, the magnetic anisotropy energy in turn reduces and consequently suppresses the coercive field. Therefore, the different bias voltages can tune the charge transfer and thereby the coercivity.

When a vdWs ferromagnet grows epitaxial on topological insulators, the magnetic properties have been demonstrated with remarkable change, due to the strong spin−orbit coupling of the topological state. Wang et al. reported a facile synthesis of Bi_2_Te_3_/FGT heterostructure (Figure 9H) by MBE.^134^ The Curie temperature of FGT was increased up to 400 K (Figure 9H), realizing the room temperature ferromagnetism. This is attributed to the interfacial exchange coupling effect, which gives rise to an increase in intralayer spin interaction in the FGT according to the theoretical calculation. A similar physical phenomenon was observed in the heterostructure of Bi_2_Te_3_/CGT.^135^ A large anomalous Hall effect was observed in Bi_2_Te_3_/CGT and persisted up to 100 K, which is large than that of pure CGT (61 K). More interestingly, the combination of 2D magnetic materials and topological insulators is expected to achieve the quantum anomalous Hall effect. A recent first-principles calculation work^136^ indicates the formation of a large exchange gap in the Te-based heterostructure MnBi_2_Te_4_/Bi_2_Te_3_. Recently, Deng et al.^137^ experimentally observed the quantum anomalous Hall effect in a MnBi_2_Te_4_/Bi_2_Te_3_ superlattice at high temperatures. Inspired by twisted graphene, the twist-stacking magnets can lead to different interlayer magnetic ordering, which provides an additional way to tune the magnetism of a 2D system. The moiré stacked bilayer CrI_3_ has been demonstrated with distinct magnetic properties from the original bilayer CrI_3_. 2D superconductor/ferromagnet interface based on all-vdWs heterostructures is expected with the exotic quantum phenomenon in the future.

## Device applications

### Magnetic tunneling junction (MTJ)

**Figure 10:**
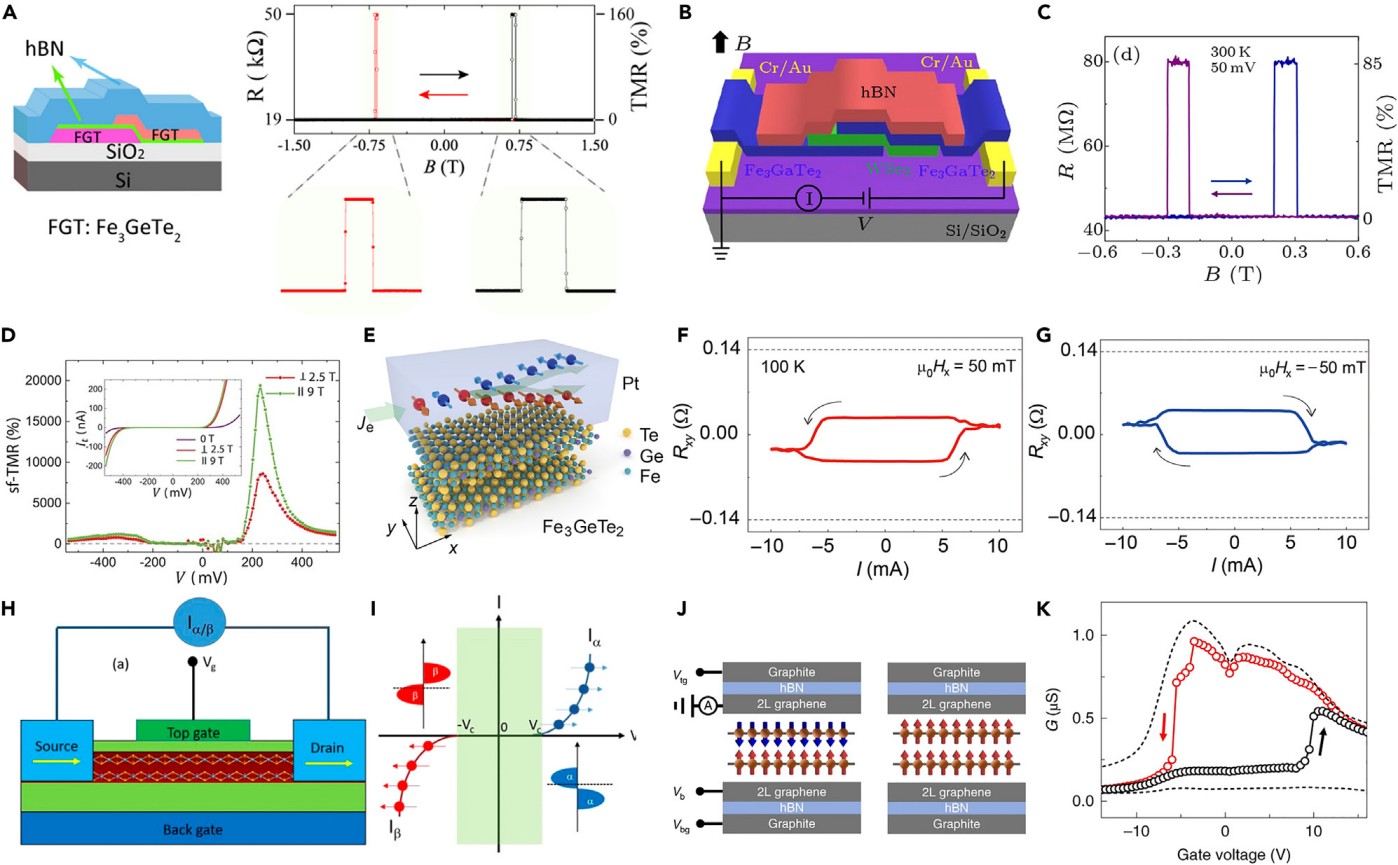
Spintronics devices based on 2D vdWs magnets (A) The schematic image and TMR effect of FGT/BN/FGT device. Reproduced with permission from Wang et al.^139^ Copyright © 2018, American Chemical Society. (B) The schematic diagram of the Fe_3_GaTe_2_/WSe_2_/Fe_3_GaTe_2_ device. (C) The 85% TMR of Fe_3_GaTe_2_/WSe_2_/Fe_3_GaTe_2_ device at room temperature. (B and C) are reproduced with permission from Zhu et al.^146^ Copyright © 2022, IOP Publishing. (D) The tunneling MR of graphene/CrI_3_/graphene devices. Reproduced with permission from Song et al.^98^ Copyright © 2018, Spring Nature. (E) The schematic image of Pt/Fe_3_GeTe_2_ device. (F and G) The magnetization reversal of FGT driven by currents under the magnetic fields of ±50 mT. (E–G) are reproduced with permission from Wang et al.^153^ Copyright © 2019, American Association for the Advancement of Science. (H) The schematic structure of the proposed spin-FET based on 2D VSe_2_. (I) The schematic spin-polarized current vs. the gate voltage of VSe_2_-based spin-FET. (H and I) are reproduced with permission from Gong et al.^160^ Copyright © 2018, National Academy of Sciences. (J and K) Tunnel FET based on graphene/CrI_3_ heterostructures. (G) Tunnel conductance of a TFET with a four-layer CrI_3_ as a tunnel barrier. (J and K) are reproduced with permission from Jiang et al.^161^ Copyright © 2019, Spring Nature.

MTJ is mainly based on vertical magnetic heterostructures, which can be simply divided into two types. The first one is the non-magnetic materials sandwiched between metallic ferromagnetic layers, also called spin valves. This effect is related to the spin-dependent scattering of electrons. When the spin orientations of the two ferromagnetic layers are the same, the spin-related scattering of the carriers is the smallest, showing a low resistance state. When the spin orientations of the two ferromagnets are antiparallel, spin-dependent scattering is enhanced, leading to a high resistance state. The magnetoresistance (MR) ratio is a critical parameter to judge the efficiency of spin-dependent electron transport in the MTJ devices. MR is defined as

(Equation 2) $$MR=R/\;R=\left(R_{AP}-R_{P}\right)/R_{P}$$

where the distinct *R*_P_ and *R*_AP_ represent the resistance measured in the parallel and antiparallel alignments of two ferromagnetic magnetizations, respectively. The first 2D vdWs spin valve dates back to 2017.^138^ Thin layers of Cr_1/3_TaS_2_ and Fe_1/4_TaS_2_ are used as magnetic metal layers, and naturally oxidized Ta_2_O_5_ on the surface of Fe_1/4_TaS_2_ is used as a tunneling insulation layer. A tunneling resistance of 13% has been achieved in this device. Then, a significantly large tunnel magnetoresistance (TMR) of about 160% at 4.2 K has been found in the FGT/BN/FGT device as shown in Figure 10A.^139^ The top and bottom FGT are prepared with different thicknesses to possess various coercive fields. Later, other 2D semiconductors are used as space layers such as MoS_2_,^140^ WSe_2_,^141^ WS_2_,^142^ and InSe.^143^ Zhu et al. reported a large MR of 41% in the 2D FGT/InSe/FGT vertical spin-valve device with applied currents below 0.1 μA at 10 K. Wang et al. studied the spin valve effect in FGT/WSe_2_/FGT with different thicknesses of WSe_2_. The TMR crossover from positive (up to +25.8%) to negative (−4.3%) with increasing the thickness of WSe_2_, which is attributed to the different symmetry-related carriers filtering for spin-polarized electrons. A similar investigation by varying the thickness of the sandwiched MoS_2_: Nb layer was reported in the Fe_3_GeTe_2_ (FGT)/Nb-doped MoS_2_ (MoS_2_: Nb)/FGT hetero-structures.^144^ Three different types of spin valves are observed, which depend on the different functionality of the spacer layers. Moving further, other vdWs metallic ferromagnetic layers served as the electrodes. Gao et al.^145^ fabricated a non-encapsulated Cr_1−x_Te/Al_2_O_3_/Cr_1−x_Te vertical spin valve device and realized a high MR ratio of 28% at 2 K. More strikingly, Fe_3_GaTe_2_ is identified as a room temperature ferromagnet and a large room temperature magnetoresistance of up to 85% has been realized based on Fe_3_GaTe_2_/WSe_2_/Fe_3_GaTe_2_ MTJ^146^ (Figures 10B and 10C), indicating the great potential in practical spintronics application.

In the second type of MTJ, the 2D vdWs magnetic materials are served as tunneling barriers, and graphene is usually used as electrodes. The tunneling MR is dominated by the spin orientation of tunneling barrier layers. Thus, this typical MTJ is also called the spin filter. Xu’s research group built a sandwich-like structure of graphene/CrI_3_/graphene spin filtering magnetic tunneling junction as shown in Figure 6D.^98^ This typical device employs the parallel/anti-parallel state of electron spin orientation in the tunneling layer CrI_3_ to regulate the flow of tunneling electrons, thus realizing the information encoding of “0” and “1”. It is worth noting that, unlike traditional magnetic tunnel junctions, the top and bottom layers of graphene are not ferromagnetic conductors, and the “tunneling MR effect” is entirely achieved by the 2D magnetic insulator CrI_3_. In other words, each layer of CrI_3_ is an independent tunneling barrier, and its magnetism determines the spin direction of the electron, thus achieving spin filtering. It is found that the tunneling MR effect increases significantly with the increase of the thickness of CrI_3_, and the MR of the four layers of CrI_3_ tunneling junction can be as high as 19000% (Figure 10D). In addition to CrI_3_, CrCl_3_^147^ and CrBr_3_^148^ are also demonstrated as function tunnel barriers to design spin-filter MTJ. The magnon-assisted tunneling was found in Gr/CrBr_3_/Gr heterostructures.^148^ Beyond the laboratory work, great progress has been made in the theoretical works of MTJ for other 2D magnetic materials. CrSBr is a typical 2D A-type AFM semiconductor. Impressively, Liu et al.^149^ calculated the TMR effect based on Gr/CrSBr/Gr structures and found TMR values are above 330%, 2 × 10^7^%, and 10^5^% with two-, four- and six-layer CrSBr at zero bias. Moreover, the theoretical results show that when 2D antiferromagnetic CrPS_4_ is used as the functional layer, the tunneling magnetoresistance is dependent on the odd-even effect of CrPS_4_ layers.^150^ When the layers of CrPS_4_ increase to 10 layers, the tunneling MR can reach up to 370 000%.

### Spin-orbit torque (SOT) devices

The technique of SOT utilizes the spin-orbit interaction to generate a spin current and thus switch the magnetization of magnetic materials, which can build the SOT-magnetic random-access memory (MRAM). The spin–orbit interaction can be determined as

(Equation 3) $$H_{SOI}=\eta_{SO}\vec{s}\times \vec{L}$$

Where *η*_SO_ is the spin–orbit interaction constant, $\vec{s}$ and $\vec{L}$ are the spin and orbital moments, respectively.^151^ A typical SOT device features an FM/non-magnetic (NM) material bilayers structure. When an in-plane electric current is injected into the NM layer, the spin current is generated by the SOC effect of spin Hall effect (SHE) of bulk NM or interface Rashba-Edelstein effect of FM/NM. Then the torque exerted by the pin accumulation can switch the magnetization of FM. Within the mechanism of the spin Hall effect, non-magnetic materials are generally with strong spin-orbit coupling and served as sources of spin-current, such as heavy metals (Pt, Ta, W et al.), transition-metal dichalcogenides (TMDs), and topological insulators. The switching efficiency parameter η and current density are the key parameters in SOT-based devices.

Alghamdi et al.^152^ and Han et al.^153^ investigated the SOT in the Pt/FGT heterostructures (Figure 10E). As shown in Figures 10F and 10G, the magnetization of few-layered Fe_3_GeTe_2_ can be effectively switched with the assistance of a small in-plane magnetic field. Benefiting from the vdWs atomic flat interface, the SOT efficiency of Pt/FGT has been proven to be much higher than that of traditional 3D materials. In addition to the metallic ferromagnets, SOT-driven magnetization dynamics are also demonstrated in some 2D semiconductor magnets. Ostwal et al.^154^ studied the SOT-driven magnetization switching in CGT/Ta heterostructures. It is found the out-of-magnetization of Cr_2_Ge_2_Te_6_ can be switched with the current densities as low as 5 × 10^5^ A cm^−2^ and in-plane fields of 20 mT. The early works on SOT require the external in-plane magnetic field to help the magnetization switching.

Recently, the SOTs in all-vdWs heterostructure have been experimentally studied.^155^ Investigations on SOT are further extended to the WTe_2_/FGT heterostructure. A large SOT efficiency (4.6) and low current densities of about 3.9 × 10^6^ A/cm^2^ were reported in WTe_2_/FGT heterostructures. While Kao et al.^156^ achieved the deterministic magnetic switching of FGT without the external field by utilizing the unconventional WTe_2_. Due to the absence of mirror symmetry of WTe_2_, when the current is along the *a-*axis, the strong out-of-plane anti-damping spin-orbit torque (SOT) exhibited in WTe_2_ is capable of facilitating field-free magnetization switching of FGT. Apart from the magnetization switching of ferromagnets, the SOT also can be proposed to be applied to antiferromagnetic systems. A bilayer CrI_3_/monolayer-TaSe_2_ vdWs heterostructure was put forward and theoretically predicted to generate the SOT.^157^ The bilayer CrI_3_ is antiferromagnetically coupled. The magnetization of the first monolayer of CrI_3_ was switched to parallel to that of the second monolayer in the absence of external fields. Apart from the traditional structures FM/NM (with strong SOC), SOT can also be achieved in the antiferromagnet/ferromagnet interface. Schippers et al.^158^ observed observe a large in-plane damping-like interfacial SOT in the heterostructure of NiPS_3_/Py. The torque conductivity σ_DL_ can reach ≈1 × 10^5^ ($\frac{\hbar }{2e}$)/(Ωm) at room temperature, which is comparable to that of traditional FM/NM structures. Beyond the above heterostructure, the topological insulator (TI)-based magnetic heterostructures Bi_2_Te_3_/CrTe_2_ can also create the integrated spin-orbit torque (SOT).^159^ And this SOT-based memristive device can simulate the fundamental synapse function, showing advantages including high linearity, long endurance, and integrated synapse-neuron functionality. With the emergence of more 2D magnets, more 2D SOT devices with high efficiency and low energy consumption are expected in the future.

### Spin field-effect transistor (Spin-FET)

Spin FET was first theoretically proposed by Datta and Das in 1990. The spins injected from the ferromagnetic electrode (source) enter a channel material. The spin polarization in the channel region can be regulated by applying gate voltages, and the spin polarization is finally detected at the drain end. The first spin FET using 2D magnets has been proposed in the bilayer 2H-VSe_2_ by quantum transport simulations^160^ (Figures 10H and 10I). The current on-off ratio can reach 10^4^ in the dual-gated 2H-VSe_2_, which is compared to that of MoS_2_ and black phosphorus. Further on, the device concept was experimentally demonstrated in the bilayer CrI_3_.^161^ Figure 10J presents the device diagram, which is composed of a dual-gated graphene/CrI_3_/graphene tunnel junction. In contrast to conventional spin transistors, this device relies on the electrical switching of the magnetization configurations of CrI_3_, different from the inject and detect spin current inside the channel. As shown in Figure 10K, the device delivers a large high–low conductance ratio approaching 400% by sweeping the gate voltage. More spin-FETs based on 2D vdWs magnets are expected and promote the development of future logic devices and information applications.

## Summary and prospects

We have taken a systematic summary to introduce the present research situation and rapid progress of 2D vdWs magnets. In the past few years, 2D magnets have experienced rapid development. The family of 2D magnetic crystals expanded gradually. The room temperature 2D ferromagnets also made some breakthroughs with the appearance of CrTe_2_ and Fe_3_GaTe_2_. More advanced techniques with high resolution are widely used to characterize the magnetic ground state of 2D vdWs magnets. Besides, external perturbations (such as electrostatic gating, light, strain, pressure, and proximity effects) are capable of effectively manipulating the magnetic behaviors of 2D vdW ferromagnetic materials and their heterostructures. At present, some 2D magnetic-based spin field effect transistors, spin filter tunnel junctions, and spin valve devices have been theoretically proposed and experimentally implemented, and have shown excellent performance, which is expected to provide a rich material platform for the development of spin-related devices in the future.

Although 2D magnetic materials have many excellent properties and broad development potential, there are still the following challenges. Most of 2D vdWs magnets widely reported are obtained by mechanical exfoliation from their bulk counterpart, which faces a big challenge in mass and controlled production. Moreover, some popular 2D magnets like CrI_3_, Fe_3_GeTe_2_, and CrTe_2_ are sensitive to ambient conditions. Their instability hinders the practical application. There are still many vdWs magnetic members under exploration. Future efforts are required to explore the synthesis methods of large-scale and air-stable 2D magnetic crystals with high transition temperatures and related vdWs magnetic heterostructures. This is undoubtedly one of the important research directions in the field of 2D vdWs magnetism. The recent research on 2D magnetism is mainly focused on ferromagnetic materials. The antiferromagnets are appealing recently for their absence of stray fields and ultrafast spin dynamics. More attention should be paid to searching for room temperature 2D antiferromagnets and their spin manipulation in the future.

The importance and uniqueness of intrinsic 2D vdWs magnetic materials are incontrovertible. There still exists a vast space in the field of 2D magnetic crystals in terms of coupling of multiple degrees of freedom such as dimensionality, charge, orbital character, lattice, valley, and topology. Furthermore, due to the unrivaled compatibility for heterostructure construction, great opportunities also exist to incorporate these 2D magnets into various magnetic heterostructures. Coupling spin with other degrees of freedom (e.g., superconductivity, ferroelectric, topological, and thermoelectric) enrich the functionality of the 2D electronic devices. The diversity of 2D magnets, stacking order, and twisting angle offer unprecedented possibilities for 2D magnetic heterostructures to explore exotic quantum phenomena. Topological superconductivity and topological Majorana state may exist on the edge of 2D magnetic materials based on the twisted-magnetic superlattices. Experimental realization of exotic states of matter such as intrinsic Chern insulators and quantum spin liquids is awaited in vdWs magnets. Moreover, new systems such as mixed dimensional van der Waals magnetic heterostructures are expected to evolve with fascinating physical phenomena. Combined with the flexibility of 2D materials, wearable flexible spintronic devices are also worthy of paying attention and importance.

In summary, the robust spontaneous spin polarization in the monolayer limit is undoubtedly of great scientific significance. Such advances in understanding and control of 2D magnetic crystals and emergent heterostructure devices will build fundamental blocks for low-power, high-speed, and ultra-compact spintronic devices, paving the way for data storage, information recognition and processing, smart sensors, and quantum computing applications. We believe that more advances and surprises are to come in the field of 2D vdWs magnetic crystals.
